# Genetic Dissection of Grain Yield Component Traits Under High Nighttime Temperature Stress in a Rice Diversity Panel

**DOI:** 10.3389/fpls.2021.712167

**Published:** 2021-09-28

**Authors:** Anuj Kumar, Chirag Gupta, Julie Thomas, Andy Pereira

**Affiliations:** Department of Crop, Soil, and Environmental Sciences, University of Arkansas, Fayetteville, AR, Untied States

**Keywords:** network analysis, linkage disequilibrium decay, number of spikelets per panicle, GWAS, rice, high nighttime temperature, USDA rice mini-core collection, panicle length

## Abstract

To dissect the genetic complexity of rice grain yield (GY) and quality in response to heat stress at the reproductive stage, a diverse panel of 190 rice accessions in the United States Department of Agriculture (USDA) rice mini-core collection (URMC) diversity panel were treated with high nighttime temperature (HNT) stress at the reproductive stage of panicle initiation. The quantifiable yield component response traits were then measured. The traits, panicle length (PL), and number of spikelets per panicle (NSP) were evaluated in subsets of the panel comprising the rice subspecies *Oryza sativa* ssp. *Indica* and ssp. *Japonica*. Under HNT stress, the *Japonica* ssp. exhibited lower reductions in PL and NSP and a higher level of genetic variation compared with the other subpopulations. Whole genome sequencing identified 6.5 million single nucleotide polymorphisms (SNPs) that were used for the genome-wide association studies (GWASs) of the PL and NSP traits. The GWAS analysis in the Combined, *Indica*, and *Japonica* populations under HNT stress identified 83, 60, and 803 highly significant SNPs associated with PL, compared to the 30, 30, and 11 highly significant SNPs associated with NSP. Among these trait-associated SNPs, 140 were coincident with genomic regions previously reported for major GY component quantitative trait loci (QTLs) under heat stress. Using extents of linkage disequilibrium in the rice populations, Venn diagram analysis showed that the highest number of putative candidate genes were identified in the *Japonica* population, with 20 putative candidate genes being common in the Combined, *Indica* and *Japonica* populations. Network analysis of the genes linked to significant SNPs associated with PL and NSP identified modules that were involved in primary and secondary metabolisms. The findings in this study could be useful to understand the pathways/mechanisms involved in rice GY and its components under HNT stress for the acceleration of rice-breeding programs and further functional analysis by molecular geneticists.

## Introduction

Rice (*Oryza sativa* L.) is the main food source for more than half the world population and one of the most important cereal crops after wheat, supplying 35–60% of the dietary calorie intake for an estimated three billion people worldwide (Fageria, 2007; FAO, 2009; GRISP, 2013). It is considered as the most diverse and versatile crop in the world, grown between 53°N in northeastern China to 35°S in New South Wales, Australia (Mae, 1997; Santos et al., 2003), and distributed across tropical, subtropical, and temperate regions (Vaughan, 1989) worldwide. Based on its evolutionary history, several studies have reported that there are two major classes or subspecies (*Indica* and *Japonica*) and two subclasses (*Japonica* classified further into tropical *Japonica*, temperate *Japonica*, and aromatic; *Indica* classified into *Indica* and *aus*) of rice growing around the world (Glaszmann, 1987; Ni et al., 2002; Garris et al., 2005; Huang et al., 2010; Zhao et al., 2011; McCouch et al., 2016; Kumar et al., 2017). More than 100 countries grow rice as a crop on more than 630 million ha, coming to an annual paddy rice harvest of more than 980 million tons (FAO, 2017; Laborte et al., 2017). However, rice production will still have to increase to keep up with the tremendous growth of the world population (Liang et al., 2010). By 2030, meeting future demand could be hindered by changing climate conditions, as water scarcity and the increased frequency of extreme weather events have shown negative impacts on rice yield (Satake and Yoshida, 1978; Jagadish et al., 2007, 2010; Mohammed and Tarpley, 2009a,b; Foley et al., 2011; Coast et al., 2015; Röth et al., 2016; Lesjak and Calderini, 2017).

The global mean surface air temperature has increased by 0.85°C over the period from 1880 to 2012, with this temperature being predicted to increase further by 1–3.7°C by the end of the 21st century, which will potentially increase the frequency and magnitude of heat stress events (IPCC, 2013). Under these scenarios, climate change has increased nighttime temperature more than daytime temperature in rice-growing areas worldwide (Peng et al., 2004; Elagib, 2010). High nighttime temperature (HNT) is one of the detrimental factors attributed to the decline in rice grain yield (GY) and quality year after year (Peng et al., 2004). Rice crops are highly sensitive to HNT stress at all their growth stages. However, rice plants at the reproductive stage are tremendously affected by HNT stress, leading to lower GY and poor grain quality under greenhouse and field conditions (Counce et al., 2005; Cooper et al., 2008; Jagadish et al., 2015; Kumar et al., 2017). Peng et al. (2004) reported that an increase of 1°C in nighttime temperature reduced rice GY by 10%. Similarly, it has been shown that HNT stress of 28 or 29°C gave rise to a cultivar-specific 10–20% GY decline (Shi et al., 2013; Bahuguna et al., 2017), and a more than 90% reduction was estimated when HNT stress increased to 32°C (Mohammed and Tarpley, 2009b). Based on several recent studies, HNT stress adversely affects all GY components, leading to significant reductions in total GY in rice (Jagadish et al., 2015; Kumar et al., 2017, Kumar et al., 2018).

An increase in the GY of cereal crops such as rice depends on the establishment of several component traits such as the panicle number per plant, panicle length (PL) (size), number of spikelets per panicle (NSP), seed set [number of filled grains per panicle (FGP)], and individual seed/grain size and weight (Chen et al., 2008; Gaju et al., 2014; Kumar et al., 2018, 2019). All these components, with and within the plants, compete for favorable growing conditions. Among these yield components in rice, panicle size, panicle number, and NSP are the major components characterized with the highest plasticity under favorable growing conditions and, therefore, have the highest impact in yield elaboration (Adriani et al., 2016). So far, HNT stress has been speculated to have an impact on panicle number, spikelet number (NSP) per panicle, spikelet fertility (SF) (FGP), and grain size and weight (Xu et al., 2020). Therefore, limited information is available on the effects of HNT stress on panicle size determining the final GY in rice (Shi et al., 2017). In recent years, HNT stress of 26–32°C showed negative effects on panicle development in specific genotypes/cultivars in comparison with the control treatments of 22–25°C in several studies (Cheng et al., 2009; Zhang et al., 2013; Mohammed and Tarpley, 2014). Anacleto et al. (2019) reported that the NSP, or NSP per panicle, is also a major yield component determined by panicle size, panicle density, and panicle branching in rice. Therefore, a compact panicle structure with a NSP ranging from 200 to 250, fully utilizing the available carbohydrates, can sustain stable GY in rice (Xu et al., 2020). Previously, it had been shown that the mechanism of spikelet development is vulnerable to high daytime temperature (HDT) conditions (Wu et al., 2016; Chaturvedi et al., 2017; Soda et al., 2018), however, HNT stress (4–6°C higher than control conditions) also reduces the NSP (Zhang et al., 2013; Thuy and Saitoh, 2017) in rice. Thus, panicle number, panicle size, and NSP substantially contribute to the total GY in rice (Li et al., 1998). Under HNT stress, both panicle size and NSP are more prone to significantly decrease than panicle numbers in rice (Xu et al., 2020). Furthermore, optimizing plant growth during panicle and spikelet development is extremely important to elevate GY under HNT stress in rice.

To understand the basis of elevating the GY and enhancing the heat tolerance of rice under HNT stress, dissecting the natural genetic variation widely distributed among the diverse rice accessions, where the identification of favorable alleles for GY components such as PL/size and NSP are the easiest phenotypes to quantify, could be a useful approach (Kumar et al., 2018, 2019). Several studies have been carried out to map and characterize the genetic variation conferring yield components and heat tolerance to rice under HDT stress (Xiao et al., 2011; Ye et al., 2012; Buu et al., 2014; Adriani et al., 2016; Cao et al., 2020; Xu et al., 2020; Chen et al., 2021). However, no extensive results of mapping studies are available under HNT stress until now (Xu et al., 2020; Kumar et al., 2020). Hence, to dissect and quantify the natural genetic variation in diverse rice accessions for “all the major loci” involved in GY components, it is necessary to make a genome-wide scan, such as through a genome-wide association study (GWAS), for different favorable/unfavorable loci needed for the trait and use such information for further advanced genetic analyses (Kumar et al., 2020).

Conventionally, genetic variation has been characterized using bi-allelic mapping populations in previous studies (Xiao et al., 2011; Ye et al., 2012; Buu et al., 2014; Adriani et al., 2016; Cao et al., 2020; Xu et al., 2020; Chen et al., 2021). However, to characterize and map extensive natural genetic variation in diverse populations, with the advancements in whole genome sequencing, the utilization of GWAS has now become extremely common in rice (Huang et al., 2010; Kadam et al., 2017). The most common approaches to GWAS are to utilize a diverse population, maximize the diversity of the alleles, and identify a larger number of potential quantitative trait nucleotides (QTNs)/single nucleotide polymorphisms (SNPs) associated with the target traits (Zhao et al., 2011).

In this study, to dissect and quantify the natural genetic variation in diverse populations, we reported the genetic dissection of the United States Department of Agriculture (USDA) rice mini-core collection (URMC) with the genotyping data set generated by whole genome sequencing that detected the best quality SNPs. The SNPs are most densely populated based on the number of SNPs/kb interrogated across the genomes. We explored the value of these resources for GWAS using GY components under HNT stress, such as PL and NSP, as the phenotypes. We also used several analytical techniques in plant genetics to identify the significant associations revealing QTNs/SNPs. Using these significant SNPs, we identified the putative potential QTNs/genes involved directly or indirectly in the development of panicles and spikelets expressing heat tolerance in rice. Furthermore, we showed the different advantages of populations of *O. sativa*, where we gain power by directly accounting for significantly associated alleles/SNPs from the mapping model. The results from the present study will aid and strengthen rice-breeding programs for high temperature tolerance using an SNP-based marker assisted selection and the pyramiding of the QTNs/SNPs related to the GY components and heat tolerance in elite rice cultivars of rice growing areas, especially the United States.

## Materials and Methods

### Plant Material and Growth Conditions

A panel of 190 diverse rice accessions, comprising 185 diverse rice accessions of the URMC and 5 well-studied rice cultivars for HNT response (Bengal, Kaybonnet, IRAT177, Vandana, and Nagina 22), was obtained from the USDA ARS Dale Bumpers National Rice Research Center, Stuttgart, AR, United States (Agrama et al., 2009). This collection was systematically developed from 1,794 core entries in the USDA rice collection based on both phenotypic and genotypic data and is a representative subset of more than 18,000 accessions of rice entries worldwide in the USDA rice germplasm collection (Agrama et al., 2009; Li et al., 2010). The panel of 190 diverse rice accessions, comprising of 102 *Indica* (*Indica* and *aus*), 81 *Japonica* (tropical *Japonica*, temperate *Japonica*, and aromatic), and 7 Admixture (mixed populations of *Indica* and *Japonica*) accessions, further comprising 53.68% *Indica*, 42.63% *Japonica*, and 3.68% Admixture accessions, was used in this study (Supplementary Table 1).

The seeds obtained from the USDA were used for multiplication and purification using the single seed decent (SSD) method for a season before this experiment was initiated. Staggered planting of this diverse panel was done to deal with the variation in heading days (HD) of the panel (Kumar, 2017). This was done with a sample of approximately 30 seeds from each rice accession of the panel, with the samples then being germinated in single plastic pots, of size 15 cm × 15 cm, filled with a mix of the SunGro professional potting mix (Sun Gro Horticulture Distribution, Agawam, MA, United States) and field soil (3:1), and grown in the greenhouse at the Harry R. Rosen Alternative Pest Control Center at the University of Arkansas, Fayetteville, AR, United States (Kumar, 2017). After 10 days of germination, equal-sized seedlings of each accession were transplanted in 3-gal plastic pots filled with the mixture of potting mix and field soil. The plants were then grown in the greenhouse until panicle initiation stage. For greenhouse conditions, the temperature was set to 30 ± 1°C (86 ± 1°F) during the day and 22.2 ± 1°C (72 ± 1°F) at night (Ghadirnezhad and Fallah, 2014). The light was set to a light/dark 13/11-h cycle with maximum photosynthetically active radiant (800–1,000 μmol PAR m^–2^ s^–1^) light and 60–65% relative humidity (RH) for the growth of the rice plants. The experimental design was a completely randomized design (CRD) with three replications (each replication is one plant in the pot). The plants were watered every day and fertilized with the Peter Professional soluble fertilizer (Allentown, PA, United States) containing chelated iron once a week for full vegetative growth. Plant protection methods were applied to prevent insect pests and diseases, which followed the Rosen Center greenhouse standard procedures.

### Phenotyping and High Nighttime Temperature Stress Treatment

**FIGURE 1:**
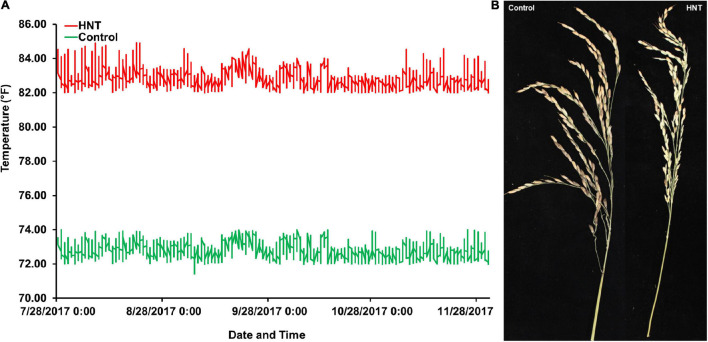
Experiment comparing high nighttime temperature (HNT) stress and control treatments on the panel of 190 diverse rice accessions of the USDA rice mini-core collection (URMC). **(A)** The nighttime temperatures record during the screen of the URMC panel. The nighttime temperature during each night was plotted across the duration of the experiment for the HNT stress and control treatments, the *red line* plots the HNT stress temperature treatment, and the *green line* is the plot of the control treatment at nighttime. The HNT stress temperature was stably maintained at 82.4 ± 1°F (28 ± 1°C), while the control treatment was held steady at 72 ± 1°F (22.2 ± 1°C) during the experiment until harvest maturity. **(B)** The effects of HNT stress compared to the control treatment on panicle length (PL) in cm and number of spikelets per panicle (NSP) in the panel.

At panicle initiation stage, as described by Counce et al. (2015), three main panicles per plant were tagged in each accession of the panel. The plants with tagged panicles were then moved to the greenhouse with the HNT stress treatment, which was maintained at a day/night temperature of 30°C (86°F)/28°C (82.4°F) for 10 h (20:00–6:00), while the control treatment was set at a day/night temperature of 30°C (86°F)/22.2°C (72°F) until harvest maturity (approximately 18–20% grain moisture content). The HOBO data loggers/sensors (Onset HOBO^®^ data logger, Cape Cod, MA, United States) were installed in both greenhouses (control and HNT stress treatments) for the continuous monitoring and recording of the day and nighttime temperatures until physiological maturity (Figure 1A). The data logger system was operated by the HOBOware^®^ Pro software/app with compatible devices. At harvesting maturity, all the panicles (under control and HNT stress treatments) for each accession of the panel were harvested separately in individual brown bags, air-dried (12–14% grain moisture content), and used for the phenotyping of GY components such as PL (cm), NSP, and many others (other components not presented here). The PL of each accession with both (control and HNT stress) treatments was measured with a plastic ruler (60 cm), while NSP of each accession was counted manually by skilled personnel.

### Statistical Analysis and Phenotype Evaluation

The statistical analysis of the phenotype data from the panel was performed using R statistical packages v3.6.2 and John’s Macintosh Project (JMP) Genomics Pro version 12.0 for descriptive statistics. The test for normal distribution and homogeneity of variances was done using the Shapiro–Wilk test and Brown–Forsythe test, respectively. An ANOVA was carried out with a statistical model that included the effects of accession, treatment (control and HNT stress), and the interaction between accession and treatment. The Tukey’s Honest Significant Difference (HSD) test was used to compare the means of treatments among all the accessions for significant effects (Tukey’s HSD, *p* < 0.05) using the *hsd* function in R packages and JMP version 12, as Tukey’s HSD can determine slight differences between the means.

In the individual and Combined genotype (*Indica*, *Japonica*, and Admixture together) populations of the panel, the Pearson’s correlation between PL and NSP for both treatments was calculated and displayed in scatter plots using the package ggplot2 in R v3.6.2. The significance of the results was tested by the function cor.test at the 95% confidence level. The means of PL and NSP in the Combined population of *Indica* and *Japonica* under HNT stress were used for the GWAS.

To quantitatively estimate the genetic variation in PL and NSP in the Combined, *Indica*, *Japonica*, and Admixture populations, the percent genetic variation (PGV) was calculated as:

$\text{PGV}=\frac{\text{Xmax}-\text{Xmin}}{\text{Xmean}}\times 100$ where the Xmax, Xmin, and Xmean are the maximum, minimum, and mean values of PL and NSP in the Combined, *Indica*, *Japonica*, and Admixture populations, respectively (Gu et al., 2014).

The broad-sense heritability (H^2^) was estimated to describe how the environment affected PL and NSP in the Combined population and the *Indica*, *Japonica*, and Admixture subpopulations using the *lmer4* function in R v3.6.2 (Bates, 2010; Bates et al., 2015a, b). The confint function (Bates, 2010) was used to compute the standard errors of the variance estimates provided by *lmer*, and these were then proliferated to use the 95% confidence intervals for the H^2^.

### Whole Genome Sequencing and SNP Detection

The total genomic DNA of each accession of the panel was extracted from fresh and early emerging young leaf tissues using the DNeasy Plant Mini Kit (Qiagen, Hilden, Germany) according to the instructions of the manufacturer and used for whole genome sequencing. All the rice genomes of the panel were sequenced by Novogene,^1^ with an average coverage of approximately 20×. The raw reads were first aligned against the reference rice genome cv. Nipponbare (IRGSP 1.0) for SNP detection. To generate the SNP dataset for the genetic dissection of the panel, 6.5 million of the most densely distributed (SNP/Kb) and high-quality SNPs, with a less than 2% missing rate and more than 5% minor allele frequency (MAF), were detected and annotated (Supplementary Figures 1A,B).

### Principle Component Analysis and Population Structure of the Panel

Principle component analysis (PCA) was performed using the Genome-wide Complex Trait Analysis (GCTA) v1.92.1 software to estimate the number of subpopulations (Price et al., 2006; Yang et al., 2011) in the panel. A population structure analysis of the panel was performed using the fastSTRUCTURE software v1.0 (Raj et al., 2014), following a Bayesian clustering approach to characterize the population structure, with the numbers of tested subpopulations (*K*) ranging from 1 to 5, and with three independent runs each. A python script “*chooseK.py*” was used to identify the model complexity that maximized marginal likelihood, choosing the most likely *K* values based on the rate of change in LnP between successive *K* values. The python script “*ditstrcut.py*” was also used to construct the population structure plot using the most likely *K* values. The marginal likelihood plot was plotted with the marginal likelihood *K* values and number of populations (*K* = 5) using the package ggplot2 in R v3.6.2.

### Linkage Disequilibrium Analysis

The genome-wide pairwise linkage disequilibrium (LD) was calculated in the Combined, *Indica*, and *Japonica* populations (the Admixture population was removed as the population size was too small) using the correlation coefficient (*r*^2^) between pairs of SNPs by using the – −*r*^2^ – -ld-win 1000 – -ld-score – -ld-score-cut-off 0.01 command in GCTA v1.92.4 beta2 (Jiang et al., 2019). A set of 6.5 million SNPs with MAF ≥0.05 was considered for LD analysis. To estimate the effect of population structure on LD decay, the LD decay was investigated using *r*^2^ values across the entire genome showing the LD decay pattern. The LD decay (*r*^2^) was plotted against the physical distance (Kb) using the package ggplot2 in R v3.6.2.

### Genome-Wide Association Studies Under HNT Stress

To identify QTNs/SNPs underlying the genetic regulation of PL and NSP in the panel, a set of 6.5 million SNPs with ≥0.05 MAF and a <2% missing rate was used for the GWAS. The genotyping (SNPs) dataset was converted to BED format using PLINK v1.9 (Purcell et al., 2007; Chang et al., 2015). The GWAS was conducted on PL and NSP GY components under HNT stress in the Combined population, and then the *Indica* and *Japonica* populations, while the Admixture population was removed as the population size was too small (less than 4% of the panel). For the GWAS, the Genome-wide Efficient Mixed Model Association (GEMMA) was the software that implemented the GEMMA algorithm (Zhou and Stephens, 2012), which uses a linear mixed model (LMM) for association tests using an estimate of relatedness matrix as a covariate. For further control of population structure, the first four principal components in the panel (Combined, *Indica*, and *Japonica* populations) were used as covariates. Genome-wide critical values were determined by permutations: each studied phenotype was permutated, and the genome-wide lowest *p*-values were recorded in the GWAS. To declare the significant associations, a threshold of −log_10_ *p* (1e−05) was set using Wald test criteria. To visualize the association results, the quantile–quantile (Q–Q) plots of observed *p*-values were constructed against expected *p*-values, and Manhattan plots were constructed with the chromosome position on the *X*-axis against −log_10_ (*p*-values) of all SNPs using the package qqman in R v3.6.2 (Turner, 2014). After the GWAS run, all the SNPs with −log_10_ > 1e−05 were considered highly significant. The highly significant SNPs associated with PL and NSP in the Combined, *Indica*, and *Japonica* populations based on their extent of LD were used to scan the reference rice genome to identify candidate genes.

### Allelic Effect and Phenotypic Variance Explained by SNP Estimation

Using the differences in means of PL and NSP between accessions under HNT stress in the Combined, *Indica*, and *Japonica* populations, GEMMA, an LMM model, was used to estimate SNP effects as the allelic effect. The SNP effect was expressed as a positive value if the allelic effect increased PL and NSP in the Combined, *Indica*, and *Japonica* populations, otherwise showing a negative value when the allelic effect decreased.

Using several variance components of the GWAS results, we estimated the proportion of variance in the phenotype explained by each SNP (PVE) using the information described by Shim et al. (2015) in equation:


$$\text{PVE}\,\left(\mathrm{SNP}\right)=\frac{2\times \left({\text{beta}}^{2}\right)\times \text{MAF}\times \left(1-\text{MAF}\right)}{\begin{array}{c} 2\times \left({\text{beta}}^{2}\right)\times \text{MAF}\times \left(1-\text{MAF}\right)+\\ \left({\left(\text{SE}\,\left(\text{beta}\right)\right)}^{2}\right)\times 2\times \text{N}\times \text{MAF}\times \left(1-\text{MAF}\right) \end{array}}$$

Where, N represents the sample size of the panel, beta is the effect for the genetic variant (SNP) of interest, SE (beta) is the standard error of effect for the genetic variant (SNP) of interest, MAF is the minor allele frequency for the genetic variant (SNP) of interest.

### Network Analysis

Statistically significant SNPs (*p* < 0.001) from the PL and NSP GWAS datasets were mapped to the rice genome (MSU v7). The SNPs that mapped within the defined genic regions for the reference genome were selected, with the rest being filtered. Note that we did not use any LD to associate genes with SNPs. This allowed us to keep the gene-list to a minimum size for downstream network analysis. Both of the trait-associated gene-lists were then used as two different queries to probe the rice regulatory network we recently created (Gupta et al., 2020). All edges that were found between the query genes were retained and grouped along with their module annotations, depicting enriched pathway annotations from Mapman, riceGYC, KEGG, and Gene Ontology Biological Processes. The resulting networks along with all the attributes were visualized in Cytoscape v3.1.

## Results

### Phenotypic Variation, Heritability Analysis, and Trait Correlation

In this study, to investigate the effects of HNT stress compared to control treatment in the panel (Figure 1A), the phenotypic variation in the *Indica*, *Japonica*, Combined, and Admixture populations were analyzed for PL and NSP yield components. The phenotypic variation in PL and NSP under HNT stress compared to control treatment is shown in Figure 1B. A wide range of the values for PL and NSP were observed in the *Indica*, *Japonica*, Combined, and Admixture populations under control and HNT stress treatments. The means, standard deviations (SDs), minimum values, maximum values, and coefficients of variation (CV) of PL and NSP in all the rice populations are summarized in Table 1. A large range of variation, explained by the PGV, was observed in the Combined population, showing 109.02% genetic variation in PL and 211.31% genetic variation in NSP under HNT stress, with 89.815% genetic variation in PL and 185.078% genetic variation in NSP in the control treatment. For PL, the *Japonica* population showed the highest PGV, which contained 102.42% genetic variation under HNT stress and 97.02% genetic variation under the control treatment, compared to the *Indica* population, which exhibited 86.42% genetic variation under HNT stress and 63.48% genetic variation under the control treatment. The Admixture population exhibited the lowest PGV, expressing 73.28% genetic variation under HNT stress and 58.21% genetic variation under control treatment (Table 1). Comparing the natural genetic variation for NSP in all the rice populations, the *Japonica* population showed the highest PGV containing 199.30% genetic variation under HNT stress and 175.71% genetic variation under the control treatment, followed by the *Indica* population exhibiting 163.19% genetic variation under HNT stress and 173.94% genetic variation under the control treatment; in comparison, the Admixture population showed 93.29% genetic variation under HNT stress and 103.89% genetic variation under the control treatment (Table 1).

**TABLE 1:** Natural variation and broad sense heritability (H^2^) in the panel of 190 diverse rice accessions of the USDA rice mini-core collection (URMC) comprising Combined, *Indica*, *Japonica*, and Admixture populations for panicle length (PL-cm) and number of spikelets per panicle (NSP) under high nighttime temperature (HNT) stress and control treatments.

Trait	Population	N^a^	Treatment	Min^b^	Max^c^	Mean	SD^d^	CV^e^	PGV^f^	H^2^
PL (cm)	Combined	190	Control	14.38	36.25	24.35	1.55	6.36	89.81	0.809
			HNT	10.56	33.02	20.6	2.00	9.70	109.02	0.698
	*Indica*	102	Control	15.74	30.92	23.91	1.46	6.10	63.48	0.778
			HNT	10.84	28.66	20.62	1.97	9.55	86.42	0.689
	*Japonica*	81	Control	14.38	36.25	22.54	1.68	7.45	97.02	0.823
			HNT	11.94	33.02	20.58	2.03	9.86	102.42	0.701
	Admixture	7	Control	14.44	28.5	24.15	1.36	5.63	58.21	0.907
			HNT	10.56	25.54	20.44	2.02	9.88	73.28	0.806
NSP	Combined	190	Control	41.8	247.2	110.98	14.16	12.75	185.07	0.827
			HNT	15.0	172.2	74.39	14.05	18.88	211.31	0.703
	*Indica*	102	Control	41.8	247.2	118.08	14.18	12.00	173.94	0.839
			HNT	15.0	137.2	74.88	13.45	17.96	163.19	0.719
	*Japonica*	81	Control	49.0	228.6	102.21	14.09	13.78	175.71	0.794
			HNT	22.2	172.2	75.26	15.41	20.47	199.30	0.675
	Admixture	7	Control	60.0	165.2	101.26	14.4	14.22	103.81	0.84
			HNT	32.2	86.2	57.88	8.31	14.35	93.29	0.821

*^*a*^Population size, ^*b*^Minimum values, ^*c*^Minimum values, ^*d*^Standard deviation, ^*e*^Coefficient of variation, ^*f*^Percent genetic variation (described in section “Materials and Methods”).*

For the broad sense heritability (H^2^) analysis in PL, the Combined population showed an H^2^ of 0.689 under HNT stress and 0.8 under the control treatment, while the *Japonica* population exhibited the highest H^2^ of 0.823 under HNT stress and 0.701 under control when compared with the *Indica* population under both treatments (Table 1). For NSP, the Combined population showed an H^2^ of 0.703 under HNT stress and 0.827 for the control treatment, while the *Indica* population showed a higher H^2^ of 0.719 under HNT stress and 0.839 under control when compared to the *Japonica* population (Table 1).

**FIGURE 2:**
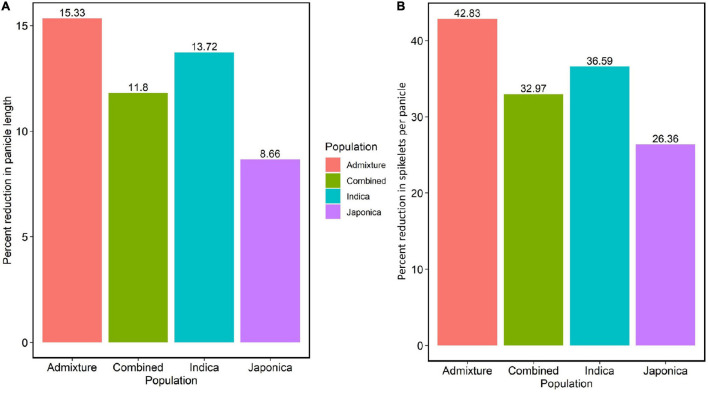
The effect of HNT stress on PL in cm and NSP in the panel of 190 diverse rice accessions of the URMC. **(A)** Percent reduction in PL in the Admixture, Combined, *Indica*, and *Japonica* subpopulations under HNT stress. **(B)** Percent reduction in NSP in the Admixture, Combined, *Indica*, and *Japonica* subpopulations under HNT stress.

To evaluate the effects of HNT stress in the experiment, we analyzed the variation of the average PL, average NSP, and their percent reductions in the Combined, *Indica*, *Japonica*, and Admixture populations. The HNT stress showed a significant effect on all populations (Supplementary Figures 2A–D) compared to the control treatment. In the study, the *Japonica* population showed the least reduction (8.66%) in PL, followed by the Combined (11.8%), *Indica* (13.72%), and Admixture (15.33%) populations (Figure 2A). Moreover, HNT stress exhibited a significant effect on NSP in all the populations (Supplementary Figures 3A–D), where the *Japonica* population showed the least reduction in NSP (26.36%), followed by the Combined (32.97%), *Indica* (36.59%), and Admixture (42.83%) populations (Figure 2B).

**FIGURE 3:**
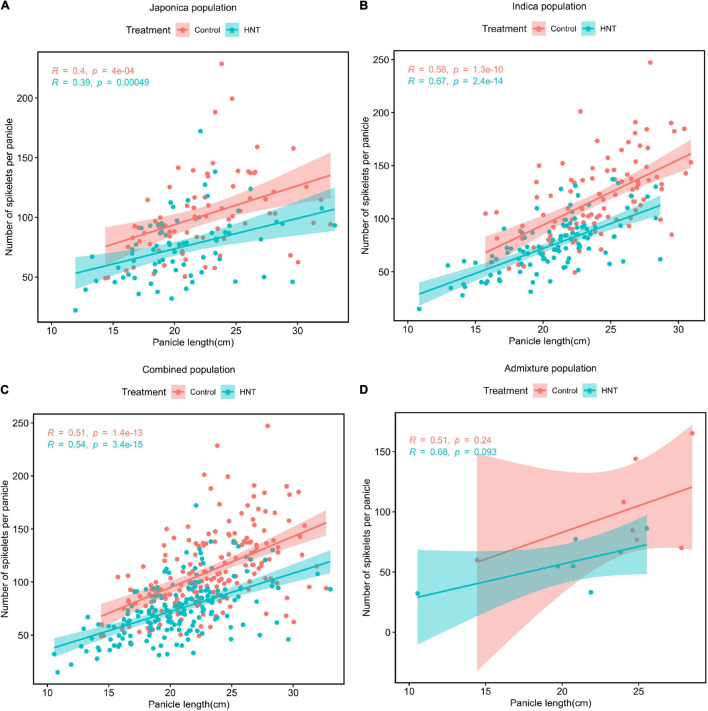
Phenotypic correlations between PL (cm) and NSP under HNT stress and control treatments in the panel of 190 diverse rice accessions consisting of the **(A)** *Japonica* population, **(B)** *Indica* population, **(C)** Combined population, and **(D)** Admixture population.

The Pearson’s correlation coefficient was determined to evaluate the relationship between PL and NSP in each of the subpopulations under the HNT stress and control treatments (Figure 3). As shown in Figures 3A–D, strong positive correlations were observed between PL and NSP under the HNT stress and control treatments in the panel, while the *Indica* population showed the highest correlation (*r* = 0.67 for HNT, and 0.58 for control) between PL and NSP, followed by the Combined (*r* = 0.54 for HNT and *r* = 0.51 for control), *Japonica* (*r* = 0.39 for HNT and *r* = 0.4 for control), and Admixture (*r* = 0.68 for HNT, and *r* = 0.51 for control) populations. The results revealed that PL showed strong positive correlation with NSP under the HNT stress and control treatments in all the populations. As expected, there was enormous natural genetic variation in the panel (the Combined, *Indica*, *Japonica*, and Admixture populations), suggesting a strong potential impact on PL and NSP for the improvement of rice cultivars in rice-growing regions under climate change conditions.

### Genetic Structure and Linkage Disequilibrium Analyses

**FIGURE 4:**
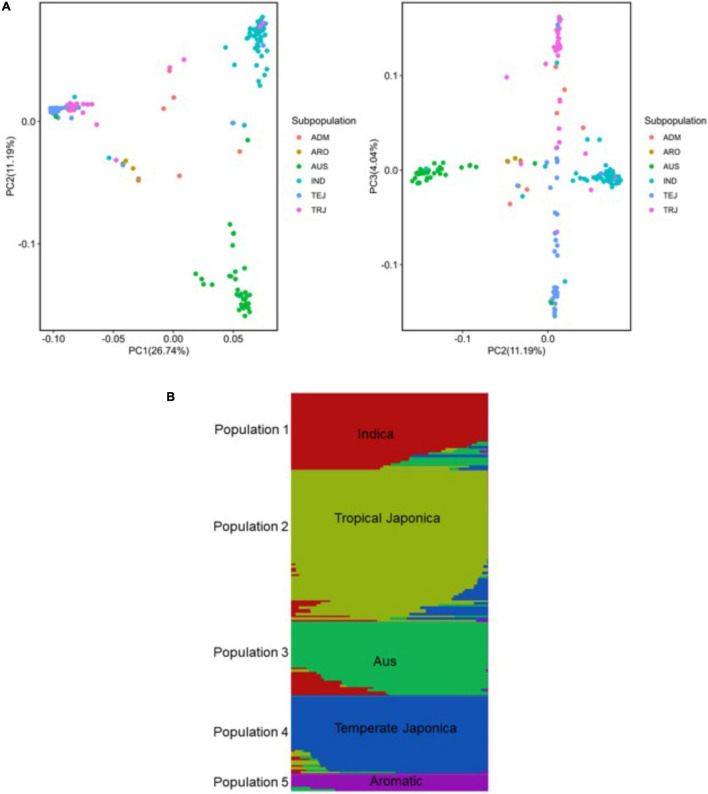
Genetic structure analysis of the panel of 190 diverse rice accessions of the URMC. **(A)** Principal component analysis (PCA) revealing global genetic variation in the panel. Left: first and second principal components (PCs); right: second and third PCs. Three PCs visualized six subpopulations in the panel. The six subpopulations, *Indica* (IND), *aus* (AUS), aromatic (ARO), temperate *Japonica* (TEJ), tropical *Japonica* (TRJ), and Admixture (ADM)-mixed subpopulations, are colored as indicated. **(B)** Population structure of the panel of 190 diverse rice accessions of the URMC; single vertical lines represent each accession and each color represents a subpopulation cluster as designated by fastSTRUCTURE. Right: the number of populations (*K* = 5) using delta marginal likelihood provided by fastSTRUCTURE and the delta marginal likelihood values are plotted against the number of populations (*K* = 5).

To dissect and characterize the global genetic variation, we applied principal component analysis (Price et al., 2006) in the URMC panel (Figure 4). The first three principle components (PCs) explained 41.97% of the global genetic variation in the panel, where the first PC split the *Indica* and *Japonica* populations in groups explaining 26.74% of the genetic variation. The second PC separated the *aus* and *Indica* subpopulations explaining 11.19% of the genetic variation (Figure 4A). The third PC separated the three *Japonica* populations into tropical *Japonica* (TRJ), temperate *Japonica* (TEJ), and *aromatic* (ARO) subpopulations with 4.04% of the genetic variation in the panel (Figure 4A).

Using a set of 6.5 million SNPs, we analyzed the population structure of the panel using fastSTRUCTURE (Raj et al., 2014). This method has been used with large sample sizes, showing a strong capability to categorize individual populations into subpopulations. For population structure analysis, the optimum number of subpopulations was determined to be *K* = 5 and the delta marginal likelihood revealed by the population structure showed optimum *K* values that categorized the panel of the URMC into the five subpopulations: *Indica*, *AUS*, tropical *Japonica*, temperate *Japonica*, and *aromatic* (Figure 4B). In the analysis, the *Indica* and *aus* accessions were represented in the *Indica* population, while the tropical *Japonica*, temperate *Japonica*, and *aromatic* accessions were included in the *Japonica* population. The results showed that the highest delta marginal likelihood was obtained when the *K* value of the panel was 1. Using the ancestry information, seven accessions were assigned into the *Admixture* population showing a mixture of *O. sativa* ssp. *Indica* (*Indica* and *aus*) with *O. sativa* ssp. *Japonica* (tropical *Japonica*, temperate *Japonica*, and *aromatic*) populations (Supplementary Table 1). The population structure analysis indicated that the panel of the URMC could be used for association analysis, as the results revealed that the natural genetic variation in the panel was widely distributed among the populations.

Linkage disequilibrium, the non-random association of alleles at different loci, indicated that genetic forces structure the genome (Slatkin, 2008). Investigations of genetic structure and LD decay patterns in the populations were the main requirements for the GWAS, with these prerequisites strengthening the interpretation of GWAS results. To maintain disequilibrium in the populations, the LD analysis was crucial to understand the decay patterns, which provide the scope for the mapping of complex traits through marker-trait associations. To determine the extent of the LD decay in all the subpopulations of the panel representing the Combined, *Indica*, and *Japonica* populations, we estimated the pairwise LD index (*r*^2^) based on the SNPs across the genome. Genome-wide LD decay patterns, along with the distance in all the populations (Combined, *Indica*, and *Japonica* populations), are shown in Supplementary Figure 4, where the *Indica* population exhibited the most rapid LD decay to 0.4 at 30 kb, followed by the Combined population LD decay to 0.4 at 120 kb, while the *Japonica* population showed the longest or most extended LD decay to 0.4 at 460 kb because of the smaller size of the *Japonica* population compared to the other populations.

### Genome-Wide Association Studies, PVE Analysis, and Putative Candidate Genes Identification

**FIGURE 5:**
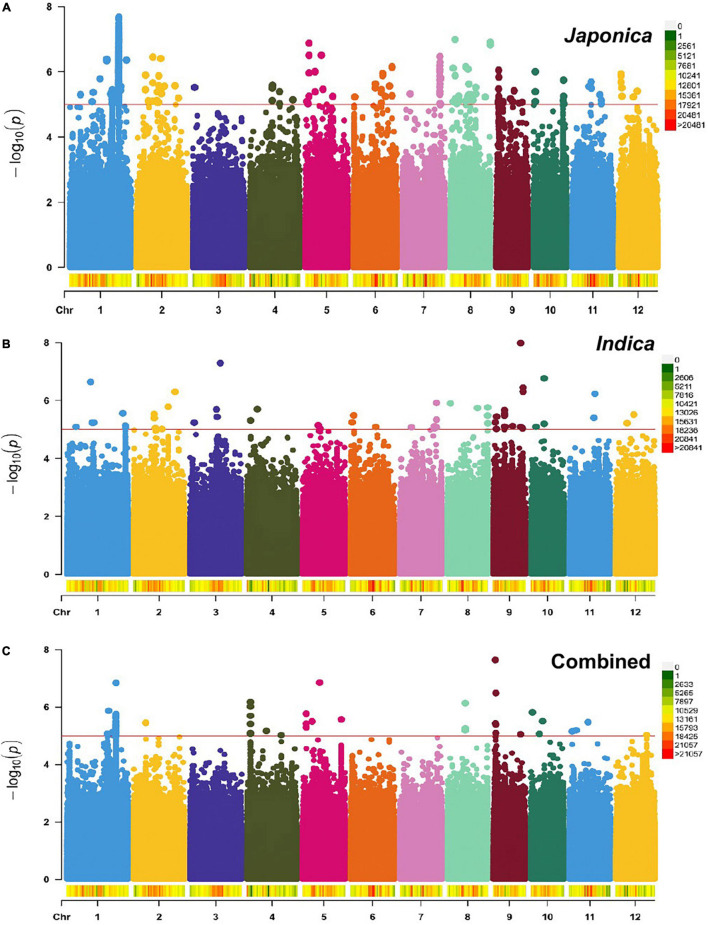
Manhattan plots showing genome-wide single nucleotide polymorphisms (SNPs) associated with PL (cm) in the *Japonica* **(A)**, *Indica* **(B)**, and *Combined* **(C)** populations, respectively, under HNT stress across the rice genomes. The *red line* represents the association threshold –log_10_ *p* ≥ 1e–05. The right-hand side scale of each plot indicates the distribution of genome-wide SNPs across the rice genomes that were used in this study.

**FIGURE 6:**
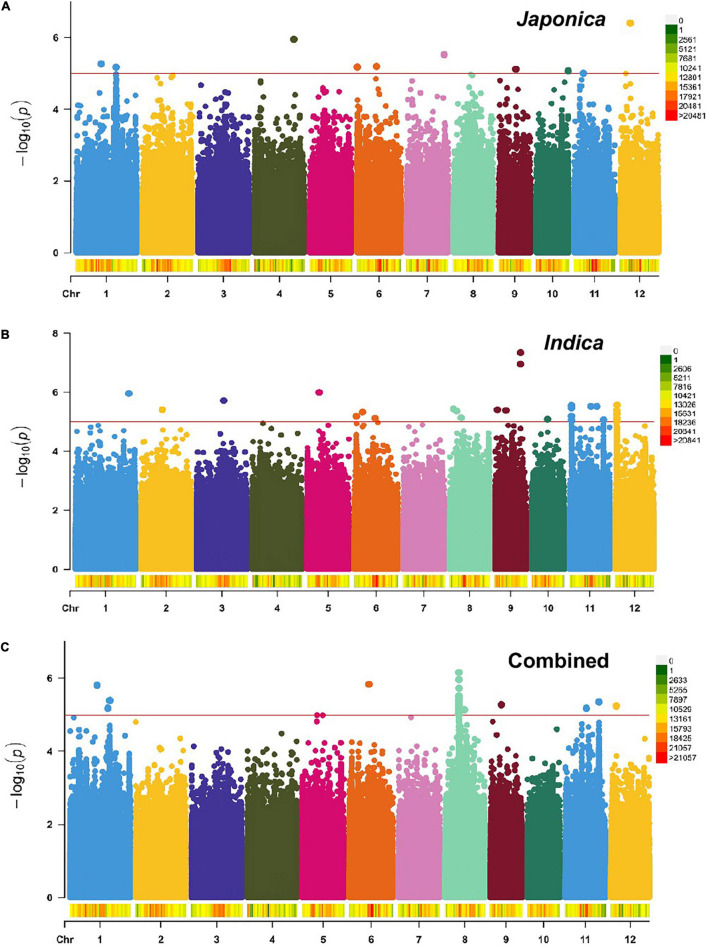
Manhattan plots showing genome-wide SNPs associated with NSP in the *Japonica* **(A)**, *Indica* **(B)**, and Combined **(C)** populations, respectively, under HNT stress across the rice genomes. The *red line* represents the association threshold –log_10_ *p* ≥ 1e–05. The right-hand side scale of each plot indicates the distribution of genome-wide SNPs across the rice genomes that were used in this study.

To identify genomic regions associated with the mapped PL and NSP traits, GWAS was carried out for the Combined population and, then, for the *Indica* and *Japonica* populations under HNT stress. To cope with false positive associations, we used a LMM, estimated the relatedness matrix as a covariate, and further controlled population structure using the first four principal components as covariates (Zhou and Stephens, 2012) in all the populations of the panel. In the Q–Q plots of PL (Supplementary Figure 5) and NSP (Supplementary Figure 6) in the Combined, *Indica*, and *Japonica* populations, the observed *p*-values followed a uniform distribution and deviated from the expected *p*-values distribution, indicating that false positives and negatives were adequately controlled. The results of the GWAS for PL and NSP in the Combined, *Indica*, and *Japonica* populations are plotted in Manhattan plots in Figures 5, 6 respectively, showing the highly significant associated SNPs above the threshold of −log_10_ *p* (1e−05).

The GWAS related to PL identified 83 highly significant SNPs (Figure 5C) showing MAF that ranged from 0.05 to 0.483 and the percent phenotypic variation explained by SNPs (% PVE) ranging from 1.39 to 12.26% in the Combined population. Of these, 60 significant SNPs (Figure 5B) exhibiting MAF ranging from 0.05 to 0.5, and % PVE ranging between 0.83 and 8.92% were in the *Indica* population and 804 significant SNPs (Figure 5A) showing MAF ranging from 0.05 to 0.5 with % PVE ranging from 0.24 to 6.93% were in the *Japonica* population (Table 2 and Supplementary Table 2). For NSP, the association analysis identified 31 highly significant SNPs (Figure 6C) with MAF ranging from 0.056 to 0.454, and % PVE ranging from 0.045 to 0.242% in the Combined population, 31 highly significant SNPs (Figure 6B) showing MAF ranging from 0.074 to 0.5 and % PVE ranged from 0.027 to 0.21% in the *Indica* population, and 11 highly significant SNPs (Figure 6A) with MAF ranging from 0.051 to 0.333, and % PVE ranging between 0.004 to 0.082% in the *Japonica* population (Table 2 and Supplementary Table 3).

**TABLE 2:** Genome-wide highly significant SNPs associated with panicle length (PL-cm) and number of spikelets per panicle (NSP) in in the panel of 190 diverse rice accessions of the USDA rice mini-core collection (URMC) comprising the Combined, *Indica*, and *Japonica* populations under high nighttime temperature (HNT) stress.

Trait	Population	N^a^	No of High Signif SNPs^b^	MAF^c^	Allelic Effect	%PVE^d^
PL (cm)	Combined	190	83	0.050–0.483	(−4.22)–4.55	1.39–12.26
	*Indica*	102	60	0.050–0.500	(−5.91)–5.74	0.83–8.92
	*Japonica*	81	804	0.050–0.500	(−3.30)–11.00	0.24–6.93
NSP	Combined	190	31	0.056–0.454	(−18.40)–24.51	0.045–0.242
	*Indica*	102	31	0.074–0.500	(−32.22)–30.69	0.027–0.21
	*Japonica*	81	11	0.051–0.333	18.86–82.59	0.004–0.082

*^*a*^Population size, ^*b*^Number of highly significant SNPs, ^*c*^Minor allele frequencies, ^*d*^Percent phenotypic variation explained by SNPs.*

The allelic effects for 83 highly significant SNPs in the Combined population, 60 significant SNPs in the *Indica* population, and 804 significant SNPs in the *Japonica* population for PL ranged from −4.22 to 4.55, from −5.91 to 5.74, and from −3.3 to 11, respectively (Table 2 and Supplementary Table 2). On other hand, for NSP, the allelic effect for the 31 significant SNPs in the Combined population, 31 significant SNPs in the *Indica* population, and 11 significant SNPs in the *Japonica* population ranged from −18.4 to 24.51, −32.22 to 30.69, and 18.86 to 82.59, respectively (Table 2 and Supplementary Table 3).

**FIGURE 7:**
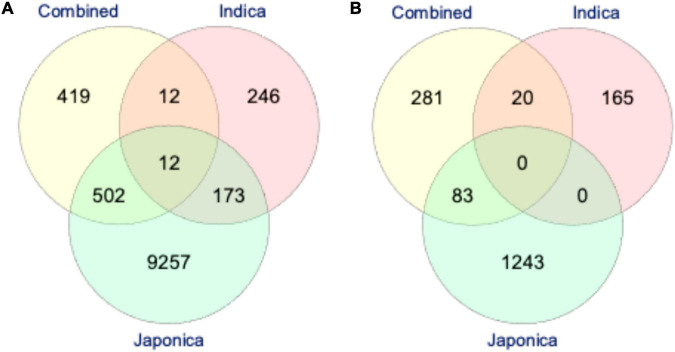
The Venn-diagram showing the number of putative candidate genes identified by highly significant genome-wide association studies (GWAS) SNPs associated with PL **(A)** and NSP **(B)** under HNT stress across the rice populations; the Combined (*yellow*), *Indica* (*pink*), and *Japonica* (*green*) populations. The reference rice genome was scanned for the identification of putative candidate genes based on the extent of linkage disequilibrium (LD) of each highly significant GWAS SNP in all the rice populations (described in “Materials and Methods”).

Based on the LD analysis, the extents of LD were 30 kb in the Combined, 120 kb in the *Indica*, and 460 kb in the *Japonica* populations. For the identification of putative candidate genes, we scanned the reference Nipponbare (*O. sativa* ssp. *Japonica*) rice genome^2^ using LD intervals of ±30, ±120, and ±460 kb of each significant SNP for PL and NSP in the Combined, *Indica*, and *Japonica* populations, respectively. For PL, we identified 945, 443, and 9,945 putative candidate genes in the Combined, *Indica*, and *Japonica* populations, respectively. On the other hand, for NSP, 384, 185, and 1,326 putative candidate genes were identified in the Combined, *Indica*, and *Japonica* populations, respectively. A Venn diagram analysis was conducted for the identification of common putative candidate genes for PL and NSP in the Combined, *Indica*, and *Japonica* populations. For PL, three sets in Figure 7A represent 419, 246, and 9,246 genes unique to the Combined, *Indica*, and *Japonica* populations. The Combined and *Indica* population sets shared 12 common putative candidate genes, the *Indica* and *Japonica* populations sets shared 173 genes, the *Japonica* and Combined population sets shared 502 genes, and the three sets together (Combined, *Indica*, and *Japonica*) shared 12 common putative candidate genes. For NSP, the three sets shown in Figure 7B represent 281, 165, and 1,243 genes unique to the Combined, *Indica*, and *Japonica* populations, with no genes common to all three populations. There were, however, 83 genes shared between the Combined and *Japonica* populations and 20 genes shared between the Combined and *Indica* populations.

### Co-localization of Significant GWAS SNPs With Previously Reported QTLs

To validate the GWAS-identified SNPs associated with the HNT responsive traits, we investigated the co-localization of all the identified highly significant SNPs and individually associated them with PL and NSP in the rice population subsets in the panel and with previously reported QTLs related to GY components under heat stress. So far, there has been no mapping study under HNT stress reported. However, several mapping studies in bi-parental populations for GY components under HDT stress have been reported (Xiao et al., 2011; Ye et al., 2012; Buu et al., 2014; Zhao et al., 2016; Shanmugavadivel et al., 2017; Zhu et al., 2017; Li et al., 2018; Cao et al., 2020; Nubankoh et al., 2020; Chen et al., 2021). The publicly available datasets of these 10 independent mapping studies published between 2011 and 2021 reported 32 major effect QTLs of GY components, such as daily flowering time (DFT), FGP, flag leaf length (FLL), GY, HD, NSP, plant height (PH), PL, panicle neck length (PNL), pollen shedding (PS), SF, and spikelet sterility (SS) under HDT stress, were downloaded and used for extraction of the genome position information of the QTL for a co-localization analysis with the significant GWAS SNPs from our analysis (Supplementary Table 4). Among all the highly significant GWAS SNPs, 140 putative candidate SNPs in the Combined, *Indica*, and *Japonica* populations associated with PL and NSP were found coincident with the genomic regions of the previously reported 32 QTLs of DFT, FGP, FLL, GY, HD, NSP, PH, PL, PNL, PS, SF, and SS GY components under HDT stress (Supplementary Figure 7 and Supplementary Table 4). Out of the 140 SNPs, 11 SNPs of the *Indica*, and 119 SNPs of the *Japonica* populations for PL were aligned over the genomic regions of these related QTLs in the rice genome, while three SNPs of the Combined, four SNPs of the *Indica*, and three SNPs of the *Japonica* populations for NSP were coincident with the genomic regions of QTLs of NSP, FGP, SF, and DFT (Supplementary Figure 7) in the rice genome.

### Network Analysis of GWAS

Next, we sought to investigate the pathways and biological processes that were potentially enriched in our GWAS study. We mapped the statistically significant SNPs to the genic regions of the reference rice genome (MSU v7) and extracted 5,265 putative candidate genes for the PL loci (Supplementary Table 5) and 3,136 putative candidate genes for the NSP loci (Supplementary Table 6). Since the rice gene ontology was incomplete and annotated only a fraction of the rice genome at the time the study was conducted, we resorted to the use of functional annotation provided by the rice regulatory network GRAiN (Gupta et al., 2020), which was developed in our lab for the network-based functional analysis and annotation of the GWAS candidate gene datasets.

We initiated our analysis by selecting the top 100 SNPs (sorted based on the significant *p-*values) from both GWAS datasets. These gene-lists were then used as inputs to the GRAiN web-application^3^ for the identification of modules containing co-regulated and potentially functionally related genes. For the PL gene-list, GRAiN reported 11 modules of co-regulated genes significantly enriched within our query (Supplementary Figure 8A) (corrected *p*-value < 0.1). Co-regulated modules in the GRAiN platform indicated groups of functionally related genes that were potentially regulated by the same sets of transcription factors (Gupta et al., 2020). The GRAiN output showed that at least 4 of these 11 PL modules were comprised of stress-related genes, signaling kinases and genes involved in post-translational modifications according to the Mapman pathway annotations and the electron transport chain according to the Gene Ontology annotations of rice. In contrast, on querying the top 100 genes from the NSP gene-list, GRAiN reported only four enriched modules, but none of them were found to be enriched with any pathway or biological processes as seen in the PL GWAS query (Supplementary Figure 8B). On expanding the input NSP query to include additional genes (*n* = 200), we found six NSP modules consisting of genes involved mainly in different categories of the carbohydrate metabolism, signaling, and DNA repair pathways. The GRAiN also reported that the promoters of the genes in the PL and NSP modules were enriched with several stress- and development-related plant *cis-*regulatory (Tables 3, 4).

**TABLE 3:** Gene ontology enrichment analysis of top 100 putative candidate genes identified using significant GWAS SNPs (*p* < 0.001) associated with panicle length (cm) in the panel under HNT stress.

Module^a^	APV^b^	GO process^c^	Mapman pathway	CRE^d^
M0135	1e-05	NA	NA	abre3hva22, sure2stpat21, gt1gmscam4, pyrimidineboxhvepb1, minus314motifzmsbe1
M0187	1e-04	NA	NA	abre3hva22, sure2stpat21, aciipvpal2, gt1gmscam4, mybcoreatcycb1, pyrimidineboxhvepb1, minus314motifzmsbe1
M0203	2e-04	NA	Stress abiotic pr proteins	abre3hva22, sure2stpat21, gt1gmscam4, mybcoreatcycb1, pyrimidineboxhvepb1, wbboxpcwrky1
M0360	0.00088	NA	Protein posttranslational modification, Signaling receptor kinases legume lectin	abre3hva22, sure2stpat21, gt1gmscam4, mybcoreatcycb1, pyrimidineboxhvepb1, 23bpuasnscycb1, mybcoreatcycb1, minus314motifzmsbe1, l1boxatpdf1, polasig1
M0144	0.00481	NA	Protein posttranslational modification, Kinase receptor like cytoplasmatic kinases, RNA regulation of transcription myB domain	abre3hva22, sure2stpat21, gt1gmscam4, pyrimidineboxhvepb1, minus314motifzmsbe1
M0026	0.02313	Electron transport chain	Stress abiotc pr proteins, signaling receptor kinases leucine rich repeat xi, Protein degradation ubiquitin	abre3hva22, sure2stpat21, gt1gmscam4, pyrimidineboxhvepb1
M0358	0.03403	NA	NA	abre3hva22, sure2stpat21, gt1gmscam4, mybcoreatcycb1, pyrimidineboxhvepb1, up2atmsd, up2atmsd, arelikeghpgdfr2
M0361	0.03403	NA	NA	abre3hva22, sure2stpat21, aciipvpal2, gt1gmscam4, aciipvpal2, mybcoreatcycb1, pyrimidineboxhvepb1, 23bpuasnscycb1, lrebox2psrbcs3, aciipvpal2, ce3ososem, mybcoreatcycb1, minus314motifzmsbe1, sp8bfibsp8bib, boxcpsas1_3, boxcpsas1_2, wbboxpcwrky1, arelikeghpgdfr2
M0087	0.03736	NA	NA	abre3hva22, sure2stpat21, gt1gmscam4, pyrimidineboxhvepb1
M0303	0.07648	NA	NA	abre3hva22, sure2stpat21, gt1gmscam4, pyrimidineboxhvepb1, 23bpuasnscycb1, minus314motifzmsbe1, boxcpsas1-2, up2atmsd
M0391	0.07648	NA	NA	abre3hva22, sure2stpat21, aciipvpal2, gt1gmscam4, gcbp2zmgapc4, aciipvpal2, mybcoreatcycb1, gcbp2zmgapc4, pyrimidineboxhvepb1, gcbp2zmgapc4, minus314motifzmsbe1

*^*a*^Module ID, ^*b*^Adjusted *p-*values, ^*c*^Gene ontology process, ^*d*^*Cis*-regulatory element.*

**TABLE 4:** Gene ontology enrichment analysis of top 200 putative candidate genes identified using significant GWAS SNPs (*p* < 0.001) associated with number of spiekelets in the panel under HNT stress.

Module^a^	APV^b^	GO process^c^	Mapman pathway	CRE^d^
M0043	0	Cellular amide metabolic process, Peptide metabolic process, cellular component biogenesis, cellular response to stress, Cellular response to DNA damage, Ribosome biogenesis, RNA metabolic process, organelle assembly, Cytoplasmic translation,	DNA repair	abre3hva22, sure2stpat21, aciipvpal2, gt1gmscam4, gcbp2zmgapc4, mybcoreatcycb1, gcbp2zmgapc4, pyrimidineboxhvepb1, gcbp2zmgapc4, minus314motifzmsbe1, gcbp2zmgapc4
M0080	0	Cell localization establishment, Intracellular transport, Vesicle mediated transport, Carbohydrate metabolic processes	Signaling g proteins	abre3hva22, sure2stpat21, gt1gmscam4, mybcoreatcycb1, pyrimidineboxhvepb1, minus314motifzmsbe1
M0093	0	NA	NA	abre3hva22, sure2stpat21, gt1gmscam4, pyrimidineboxhvepb1
M0450	7e_05	NA	Signalling receptor kinases	abre3hva22, sure2stpat21, gt1gmscam4, mybcoreatcycb1, pyrimidineboxhvepb1, dre2corezmrab17, minus314motifzmsbe1, pyrimidineboxosramy1a, mycaterd1, arelikeghpgdfr2
M0256	0.00268	NA	NA	abre3hva22, sure2stpat21, gt1gmscam4, pyrimidineboxhvepb1, minus314motifzmsbe1
M0207	0.00504	NA	NA	abre3hva22, sure2stpat21, gt1gmscam4, pyrimidineboxhvepb1
M0046	0.0176	Cellular catabolic process, defense response, cell death, programmed cell death	Signalling receptor kinases	abre3hva22, sure2stpat21, gt1gmscam4, mybcoreatcycb1, pyrimidineboxhvepb1
M0070	0.0176	NA	NA	abre3hva22, sure2stpat21, gt1gmscam4, mybcoreatcycb1, pyrimidineboxhvepb1
M0112	0.08524	Membrane lipid metabolic process, Sphingolipid metabolic process	NA	abre3hva22, sure2stpat21, gt1gmscam4, mybcoreatcycb1, pyrimidineboxhvepb1, 23bpuasnscycb1, minus314motifzmsbe1, sp8bfibsp8bib
M0432	0.0941	NA	NA	abre3hva22, sure2stpat21, gt1gmscam4, pyrimidineboxhvepb1, 23bpuasnscycb1, wboxhviso1

*^*a*^Module ID, ^*b*^Adjusted p-values, ^*c*^Gene ontology process, ^*d*^*Cis*-regulatory element.*

**FIGURE 8:**
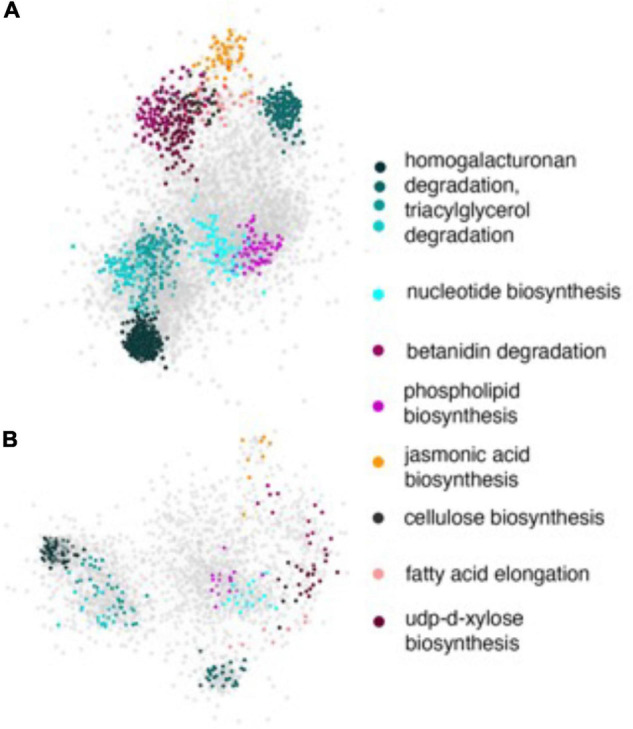
Subnetworks of PL and NSP. All statistically significant SNPs were mapped to the genic regions of the rice reference genome and the resulting genes were used as input to query the rice regulatory network. **(A)** The subnetwork induced by the PL gene-list and **(B)** the subnetwork induced by NSP gene list. In both the panels, genes are represented by ellipses and are color coded according to the module they are a part of. Gray ellipses represent genes that are part of modules with no functional annotations yet. The functional annotations of colored modules are shown in the key on the right. Note that some of these modules were not significantly enriched within the query genes.

The network enrichment analysis described above required working with small lists of genes in order to perform reliable statistical analyses. However, our GWAS identified several other genes with nominal *p-*values that could also potentially play role in other pathways and processes perturbed by HNT stress. Considering that the rice functional gene annotations were not complete during the time the study was conducted, we suspected that these pathways might not show enrichment within the statistical thresholds by GRAiN. Therefore, we probed the network with the full set of significant SNPs and extracted the subnetworks induced by all significant SNPs on both GWAS-derived datasets. On visualizing these PL and NSP subnetworks along with their attributes in Cytoscape, we observed that the PL subnetworks (Figure 8A) were considerably denser (more connections between query genes) compared to the NSP network (Figure 8B). We also observed that the subnetworks consisted of several modules enriched with genes involved in the various primary and secondary metabolism pathways relevant to the context of the GWAS. For example, we observed that a considerable number of SNPs in the PL subnetwork were involved in homogalacturonan degradation, a pathway that is believed to be modulated in stress responses. The PL subnetwork was also characterized by the presence of several biosynthetic pathways, such as for cellulose and xylose biosynthesis, fatty acid elongation, and jasmonic acid biosynthesis. These processes have been evidently linked to stress responses in plants. It must be noted, however, that some of these modules were not observed when only the highly significant SNPs were considered in the enrichment analysis, indicating that SNPs with nominal significance revealed biologically meaningful roles in the GWAS for PL determination.

## Discussion

High nighttime stress is a major environmental stress factor that negatively affects GY components and ultimately reduces GY in rice. The rice crop is highly sensitive to heat stress at every growth stage; however, the reproductive stage beginning with the early panicle development phase is extremely sensitive to such, causing a reduction in PL and NSP and subsequently reducing total GY (Shi et al., 2017; Kumar et al., 2018; Chen et al., 2021). Like other abiotic stress tolerances (drought and salinity), HNT stress tolerance is also controlled by a large number of genes and related traits. So far, only a few studies have been conducted to characterize the genetic variation in mapping populations and map the genome regions conferring GY components in rice under HDT stress (Buu et al., 2014; Adriani et al., 2016; Shanmugavadivel et al., 2017; Xu et al., 2020). Furthermore, a large genome-wide scale genetic analysis of the diverse natural genetic variation has not yet been done for GY components in rice under HNT stress (Kumar et al., 2020; Xu et al., 2020). Therefore, we first chose to survey the broad natural genetic variation in diverse rice populations and use the power of GWAS to map and compare the loci/SNPs affecting GY components in rice under HNT stress from diverse sources.

To investigate and quantify the natural genetic variation in rice, a panel of 190 diverse accessions consisting of 102 *Indica* (*Indica* and *aus*), 81 *Japonica* (tropical *Japonica*, temperate *Japonica*, and aromatic), and 7 Admixture (a mixture of *Indica* and *Japonica*) accessions were phenotyped for the GY components PL and NSP under HNT stress. The effects of HNT stress compared to the control treatment in PL and NSP were observed and quantified in the panel (Figure 1B), where the PL and NSP phenotypes of the panicles after HNT stress compared to control treatment was visually evident and efficiently quantifiable. The phenotypic variation in PL and NSP under HNT stress compared to the control treatment varied extensively among the rice accessions in the panel, which was important for genetic dissection by association mapping analysis. For PL and NSP, the Combined population revealed a wide range of genetic variation under HNT stress compared to the control treatment, with the *Japonica* population showing the highest genetic variation followed by the *Indica* and Admixture populations under HNT stress, when compared to the control treatment (Table 1). Along with the highest percentage of genetic variation, the *Japonica* population also showed the least reduction in PL and NSP under HNT stress compared to the *Indica* populations, demonstrating significantly better HNT stress tolerance. The Admixture population, on the other hand, showed the highest reduction in PL and NSP under HNT stress. This may be due to the ecosystem origins of the *Japonica* population collected from around the world, in which approximately more than 50% of the accessions were from tropical/sub-tropical regions of the globe. The broad sense heritability for PL and NSP in the *Japonica* population was moderate to higher than the Combined and *Indica* populations, while the Admixture population had even more than the other populations, which was expected because the sample size of the Admixture population was very small (seven accessions) and contained mixed genetic backgrounds representative of different ecosystems. Strong positive correlations between PL and NSP and a higher broad sense heritability for PL and NSP were found in the *Japonica* population when compared to the other rice populations in the panel, showing trait stability under HNT stress environments. This could be a good prospect for selecting the *Japonica* population to develop pure-line material for testing in multiple replications with a high frequency and the magnitude of heat stress environments. Furthermore, the identified putative candidate SNPs/loci and insights derived from the genetic dissection of the URMC panel for GY components in this study may also facilitate rapid progress in rice breeding compared to traditional approaches.

To understand and dissect the genetic architecture of the panel, the structure analysis and PCA with three first components explained approximately 42% of the global genetic variation in the panel of the URMC (Figure 4A). Similarly, McCouch et al. (2016) reported that the rice diversity panel (RDP) 1 showed 40% global genetic variation using the high-density array (HDRA) of 700K SNPs, which grouped all the individuals of each subpopulation together and separated them from other subpopulations in clusters. The findings of this study suggest that the panel demonstrated extensive natural genetic variation within the rice populations, which can provide opportunities for rice breeders and geneticists to utilize the strength of the natural genetic variation that has been well maintained in gene banks worldwide. To exploit the enormous natural genetic variation for candidate gene discovery (Garris et al., 2003) and infer evolutionary forces (Rakshit et al., 2007), it has been of intense interest to dissect the LD levels and patterns in rice populations. To investigate the LD patterns and levels in the panel, the Combined population exhibited the most rapid LD decay, followed by the *Indica* population. However, the *Japonica* population with a highly extended LD decay showed the longest extent of LD in the genome, which was possible because of the small size of the *Japonica* population in the panel. Furthermore, to contribute to the emergence and maintenance of the extents of LDs and patterns, there are several forces that can be utilized, including mutation drift, population bottlenecks, population structure and Admixture, population size, and levels of inbreeding and selection (Mather et al., 2007). In several crops and their subpopulations, LD has been characterized, in which maize exhibited the most rapid LD decay (to 0.3 at 2 kb) because of it being an out crosser (Remington et al., 2001; Tenaillon et al., 2001), while barley, a self-pollinated crop species, showed approximately 90 kb LD decay (Caldwell et al., 2006). Furthermore, Hyten et al. (2007) reported that soybean, a self-pollinated crop species, could extend from 90 to >500 kb in landrace material, even though the extent of LD depends on the population size. Like our study in rice, McCouch et al. (2016) reported that LD decayed rapidly with distance in all rice populations, with *Indica* exhibiting the most rapid LD decay and *Japonica* showing the most extended LD. Thus, insights on LD levels and patterns in rice populations determined that a modest number of SNPs could provide genome-wide coverage for association analyses.

In order to understand the origin and distribution of natural genetic variation in the rice subspecies, GWAS was performed in the Combined population and individually in the *Indica* and *Japonica* subpopulations for PL and NSP under HNT stress. The association analysis found the highest number (804 SNPs) of highly significant SNPs associated with PL in *Japonica* when compared to the Combined (83 SNPs) and *Indica* (60 SNPs) populations (Table 2 and Figures 5A–C), which explained a larger range of % PVE (from 0.24 to −6.93%) compared to other populations. This is due to the large number of polymorphic SNPs that contributed to the natural genetic variation in the population. Moreover, these highly significant SNPs of the *Japonica* population demonstrated the highest allelic effect, ranging from −3.3 to 11, compared to the other populations (Combined and *Indica*), suggesting that the *Japonica* population contained a larger repertoire of favorable variant alleles contributing to panicle development and stress tolerance under HNT stress conditions. Along with PL, the variation for NSP in the *Japonica* population showed the largest allelic effect, which was governed by highly significant SNPs that may, in turn, increase the number of spikelets in the population compared to other populations, as the development of NSP in rice directly or indirectly depends on the PL/size (Li et al., 1998). Using the extents of the LDs of these populations for each highly significant SNP associated with PL and NSP, we scanned the reference rice genome (Nipponbare) for the identification of putative candidate genes. The *Japonica* population contained the highest number of putative candidate genes (9,257 genes for PL and 1,326 genes for NSP) compared to other populations (Figures 7A,B). Interestingly, the Venn diagram analysis revealed remarkable findings, in which 12 putative potential candidate genes identified through highly significant SNPs associated with PL were found to be common in all the three rice populations (Combined, *Indica*, and *Japonica*), 173 putative candidate genes were common in the *Indica* and *Japonica* populations, and 83 putative candidate genes identified by GWAS SNPs associated with NSP were common in the *Indica* and *Japonica* populations. These common genes could be potential candidate genes for further analysis and validation in rice accessions with extreme phenotypes for high and low scores for GY components under HNT stress using transcriptome analysis and followed up by genome editing in elite rice cultivars.

The identification of highly significant SNPs co-localized with the genomic regions of earlier reported/published QTLs for GY components in rice under heat stress provided strength and evidence for true marker trait associations in rice. All the highly significant SNPs associated with PL and NSP under HNT stress were found to overlap with genomic regions of previously reported QTLs for GY components under HDT stress. Out of these, 140 highly significant SNPs associated with PL and NSP were coincident with the genomic regions of 32 previously reported QTLs related to potential GY components across the rice genome (Supplementary Table 4). The significant SNPs on chromosomes 1 and 4 showed the strongest evidence of co-localization with independently derived data from the earlier reported QTLs related to GY components, which suggests that these chromosomal regions bear useful gene targets to understand the phenomenon of heat stress in rice. There were several highly significant SNPs associated with PL and NSP that directly overlapped the QTLs regions related to PNL, PL, and number of spikelets under heat stress in rice. Chen et al. (2021) reported a fine mapping study of *qHTT8* on chromosome 8 related to heat tolerance leading to spikelet fertility in rice, and they mapped the genomic region from 3,555,000 to 4,520,00 bp (on chromosome 8 where, interestingly, three highly significant SNPs from our GWAS were overlapped in a 76-bp interval region of this QTL). In summary, the data from the comparison of 140 highly significant SNPs for the effect of HNT on rice GY and quality components and previously reported QTLs have revealed very useful information, which can be implemented in rice breeding using SNP-based marker-assisted selection and introgression of the co-localized genomic regions with these QTLs of GY into elite rice cultivars for the development of high-yielding rice cultivars under heat stress environments. In addition, a follow-up search for the SNPs we found in other germplasms might enable the selection of the trait phenotypes for other novel elite alleles.

By using the global genetic variation (approximately 42% genetic variation) and validation of GWAS results of the Combined population in the panel, GO enrichment and network analysis, with the 5,265 associated genes identified by all the GWAS SNPs associated with PL and 3,136 genes identified by all GWAS SNPs associated with NSP at *p* < 0.001, identified two modules (M028 and M023) in the rice gene network. The potential functions of these modules (programmed cell death, response to abiotic stress, and signaling kinase receptor pathways) and related CREs are all well-known stress response mechanisms of plants. The recovery of these modules by our GWAS testified for the accuracy of our study and led to new functional insights on the potential functions of the candidate genes that were selected. For instance, some of the putative candidate genes are yet to be unannotated but lie within the network modules annotated as cellular metabolic, response to stress, and carbohydrate derivative metabolism. These processes are relevant for spikelet/grain development and carbohydrate metabolism. Within the CREs, abre3hva22 is a well-known ABA-response element involved in abiotic stress responses and sure2stpat21 is a sucrose-dependent element involved in plant growth and development. Insights from GO enrichment and network analysis, thus, show the potential to enhance our understanding of the basis of mechanisms involved in heat tolerance, subsequently contributing to increases GY in rice under heat stress conditions.

## Conclusion

This is the first integrated study to screen a panel of diverse rice accessions at the panicle initiation stage for GY components such as PL and NSP under HNT stress and dissect the panel in subsets of *Indica*, *Japonica*, Admixture, and Combined subpopulations at the phenotypic and genetic level. Phenotypic screens indicated that the *Japonica* population showed the least reduction in PL and NSP under HNT stress compared to the Combined and *Indica* populations, while the *Admixture* population exhibited the highest reduction. In addition, the highest percentage of genetic variation and higher broad sense heritability in the *Japonica* subpopulation suggested that the *Japonica* population, as an exotic gene pool, could be used in rice breeding programs, especially since the United States generally grows *Japonica* rice. Trait correlation analysis determined that PL was positively correlated with NSP in the Combined, *Indica*, and *Japonica* populations under HNT stress, and, like other important GY components, panicle size and NSP were crucial components contributing to GY enhancement in rice. Using the global natural genetic variation in the populations, we found highly significant SNPs associated with PL in the Combined (83 SNPs), *Indica* (60 SNPs), and *Japonica* (804 SNPs) populations. For NSP, there were 31 highly significant SNPs in the Combined, 31 highly significant SNPs in the *Indica*, and 11 highly significant SNPs in the *Japonica* populations. Using these SNPs, we confirmed that 140 significant SNPs associated with PL and NSP were coincident with previously reported genomic regions of the QTLs of major GY components in rice under heat stress. The SNPs that were coincident with previously reported QTLs and all significant GWAS SNPs, in all the populations, could be important resources for introgression or the pyramiding of favorable alleles to improve rice cultivars for heat tolerance and GY. Enrichment and network analyses provided additional insights into the biological processes involved in pathways and CREs, which are involved in responses to abiotic stress and plant growth and development. Therefore, all the findings from this study are noteworthy, with the phenotypic and genetic dissection of the panel providing deep insights into the phenotypic and genetic variations in different subpopulations, which are also accessible to rice breeders and geneticists, to understand the mechanisms related to heat stress tolerance contributing to the stability of GY in rice.

## Data Availability Statement

The datasets presented in this study can be found in online repositories. The names of the repository/repositories and accession number(s) can be found in the article/Supplementary Material.

## Author Contributions

AK and AP conceived the idea and designed the study. AK and JT performed phenotypic screening in the greenhouse. AK and CG performed the bioinformatics analysis. AK performed the phenotypic and genotypic data analysis and drafted the manuscript. AP acquired the funding and supervised the research. All authors approved the content of this manuscript.

## Conflict of Interest

The authors declare that the research was conducted in the absence of any commercial or financial relationships that could be construed as a potential conflict of interest.

## Publisher’s Note

All claims expressed in this article are solely those of the authors and do not necessarily represent those of their affiliated organizations, or those of the publisher, the editors and the reviewers. Any product that may be evaluated in this article, or claim that may be made by its manufacturer, is not guaranteed or endorsed by the publisher.

## Footnotes

1
www.en.novogene.com

2
http://rice.plantbiology.msu.edu/

3
http://rrn.uark.edu/shiny/apps/GRAiN/
