# Supplementary material for: Genetic Dissection of Grain Yield Component Traits Under High Nighttime Temperature Stress in a Rice Diversity Panel
Source: Front Plant Sci. 2021 Sep 28;12:712167. doi: 10.3389/fpls.2021.712167 (PMC8508263; doi:10.3389/fpls.2021.712167)
Supplement: Supplementary file 1 [file Data_Sheet_1.pdf]

A

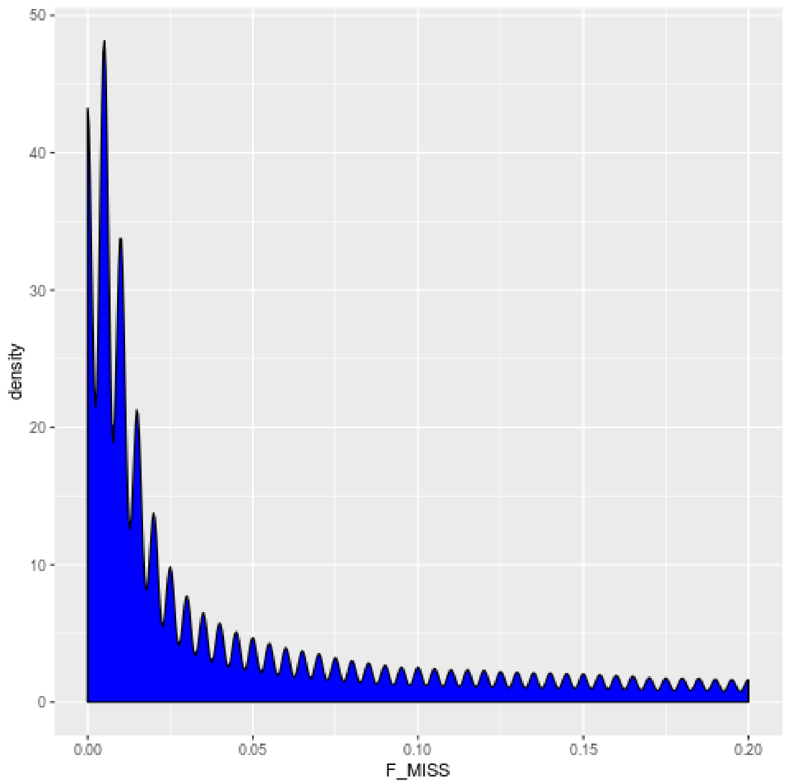

B

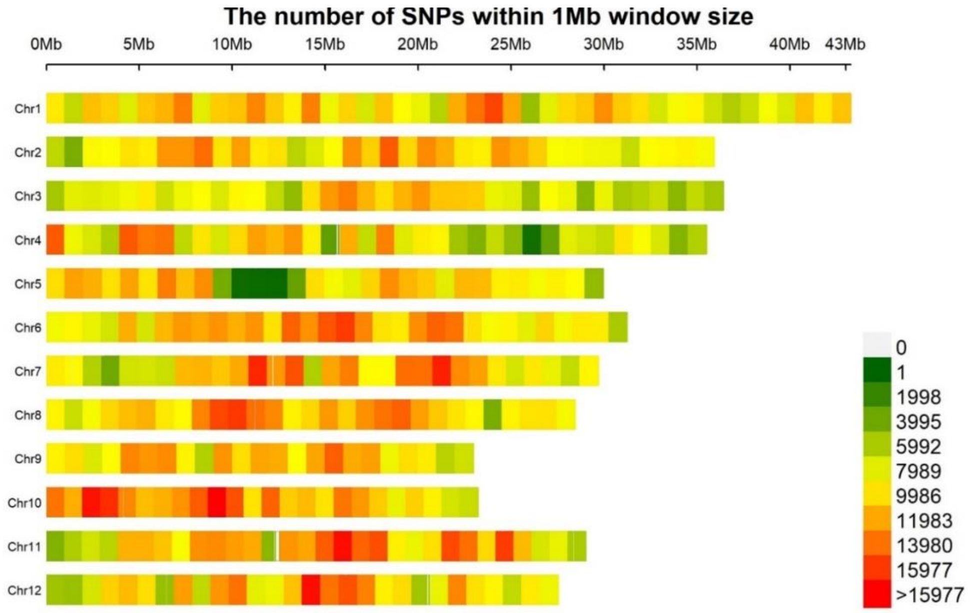

Supplementary Figure 1| Highly significant and dense aggregate of single nucleotide polymorphisms (SNPs) in the panel of 190 diverse rice accessions. The SNPs were detected by the alignments of raw reads of 190 rice genomes with the reference Nipponbare rice genome. **(A)** Missing rate of SNPs showing less than 2% missing SNPs, and the SNP density were plotted against the fraction of missing SNPs on the rice chromosomes. **(B)** Distribution of genome-wide SNPs within a 1 Mb sliding window on the 12 rice chromosomes of the rice genomes.

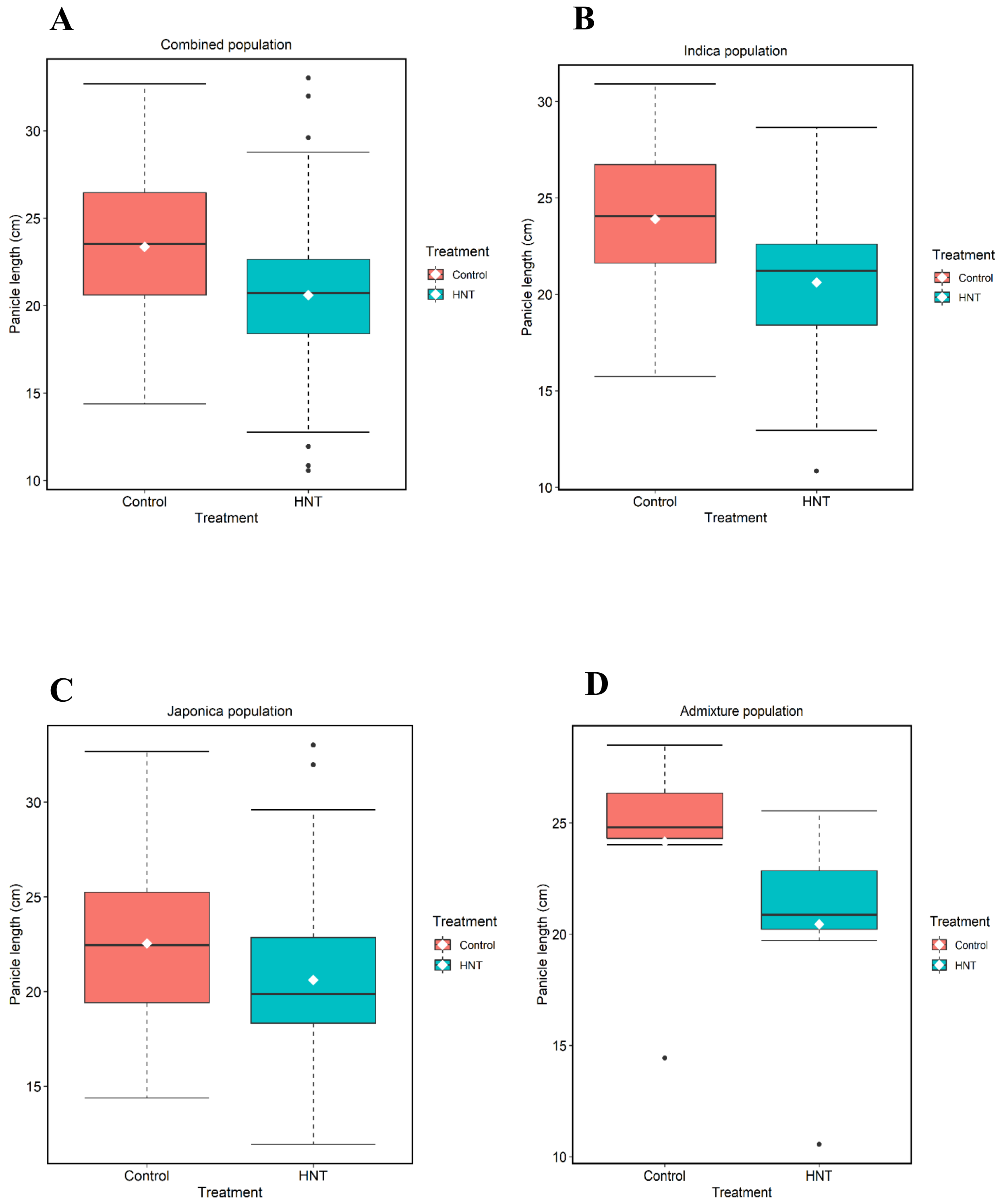

Supplementary Figure 2| The box plots show the effects of high nighttime temperature (HNT) stress compared to control treatment on panicle length (cm) in the populations: **(A)** Combined, **(B)** *Indica*, **(C)** *Japonica*, and **(D)** Admixture populations.

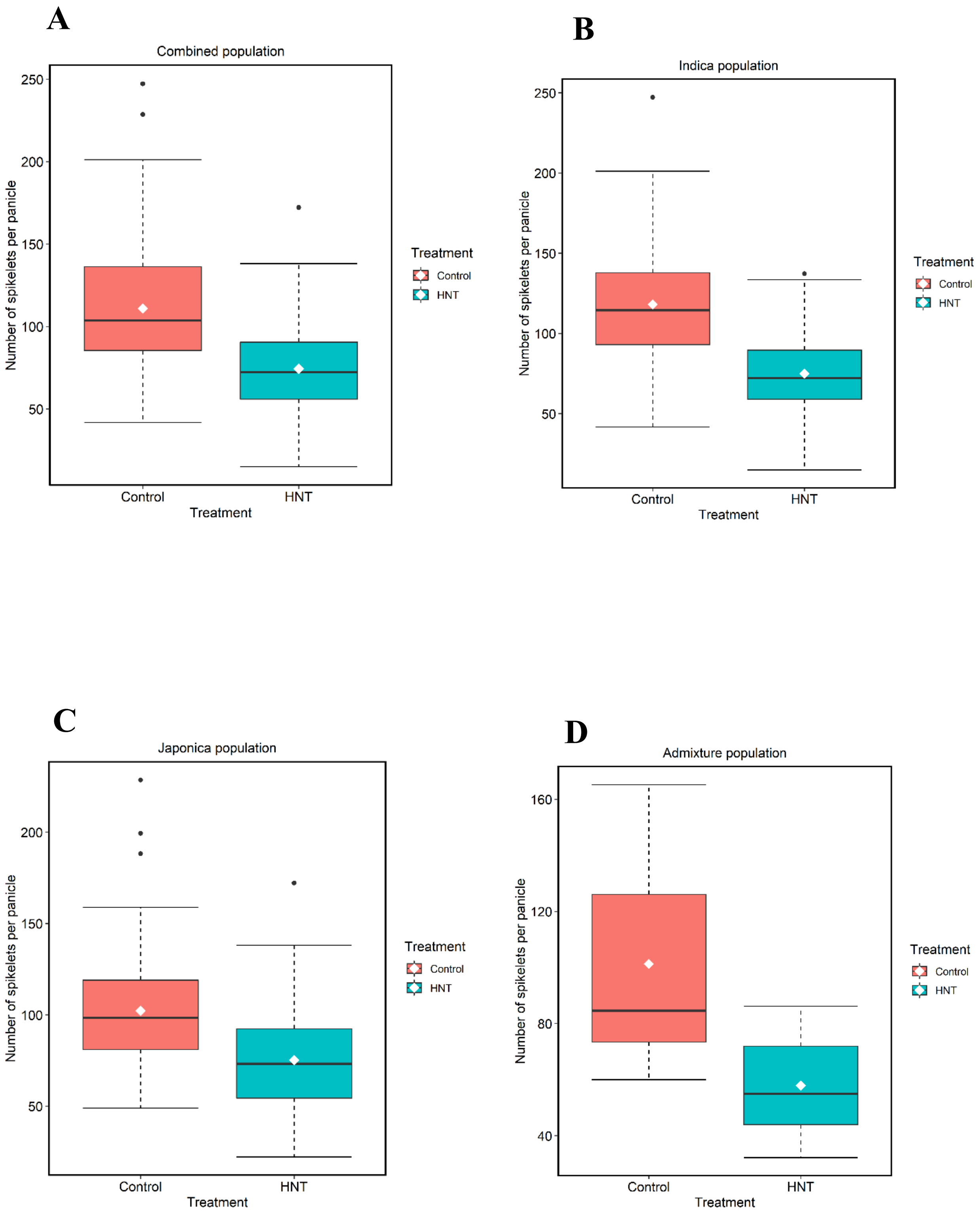

Supplementary Figure 3| The box plots show the effects of high nighttime temperature (HNT) stress compared to control treatment on number of spikelets per panicle in the **(A)** Combined, **(B)** *Indica*, **(C)** *Japonica*, and **(D)** Admixture populations.

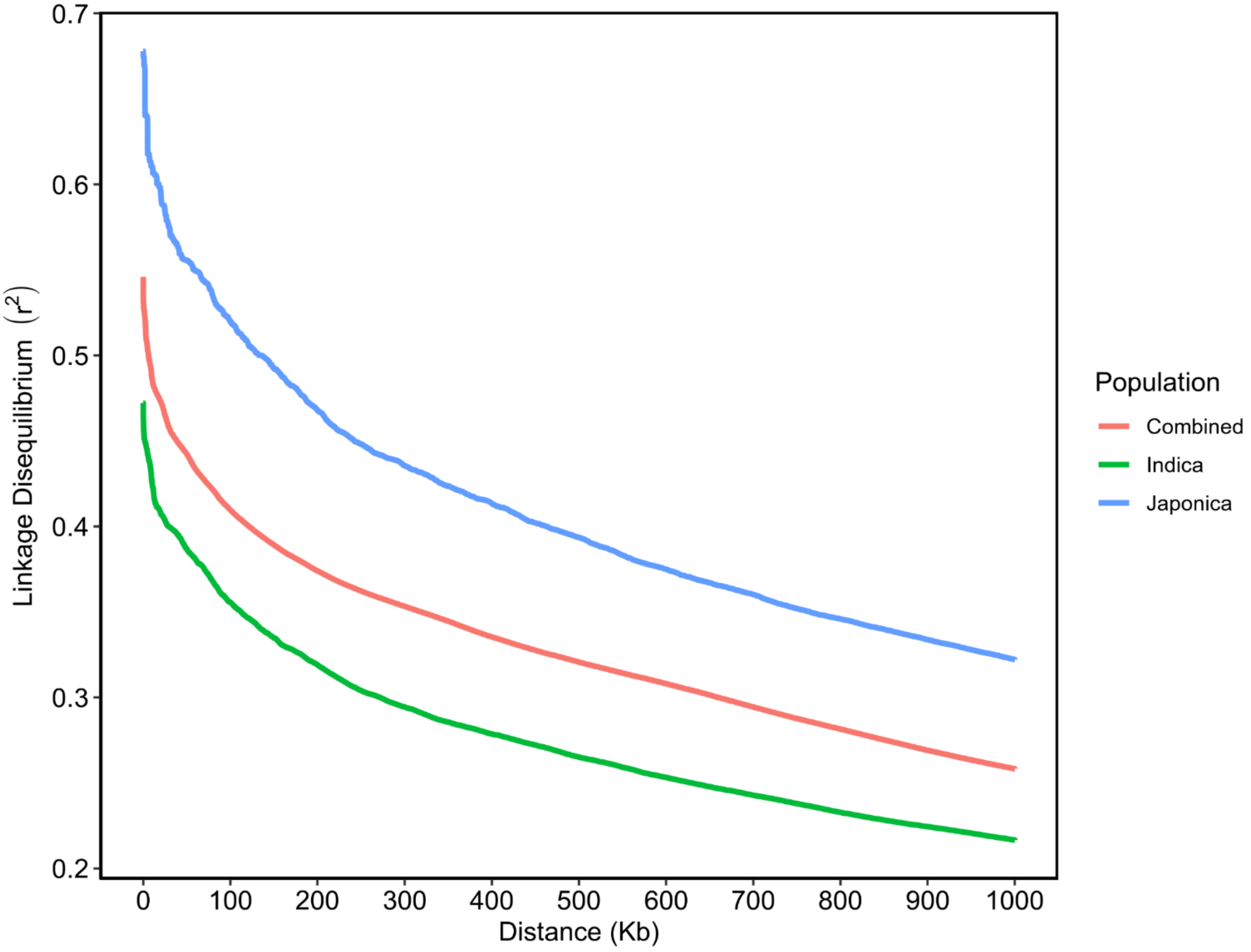

Supplementary Figure 4| Comparison of linkage disequilibrium (LD) patterns and LD decay levels in the Combined, indica, and japonica populations in the panel. Genome-wide average LD decay was estimated in all the rice populations in which decay of LD (r^2) was plotted against distance of SNPs in the rice genome. LD decay plot represents patterns and levels of LD decay in the combined (pink line), indica (green line), and japonica (blue line) populations.

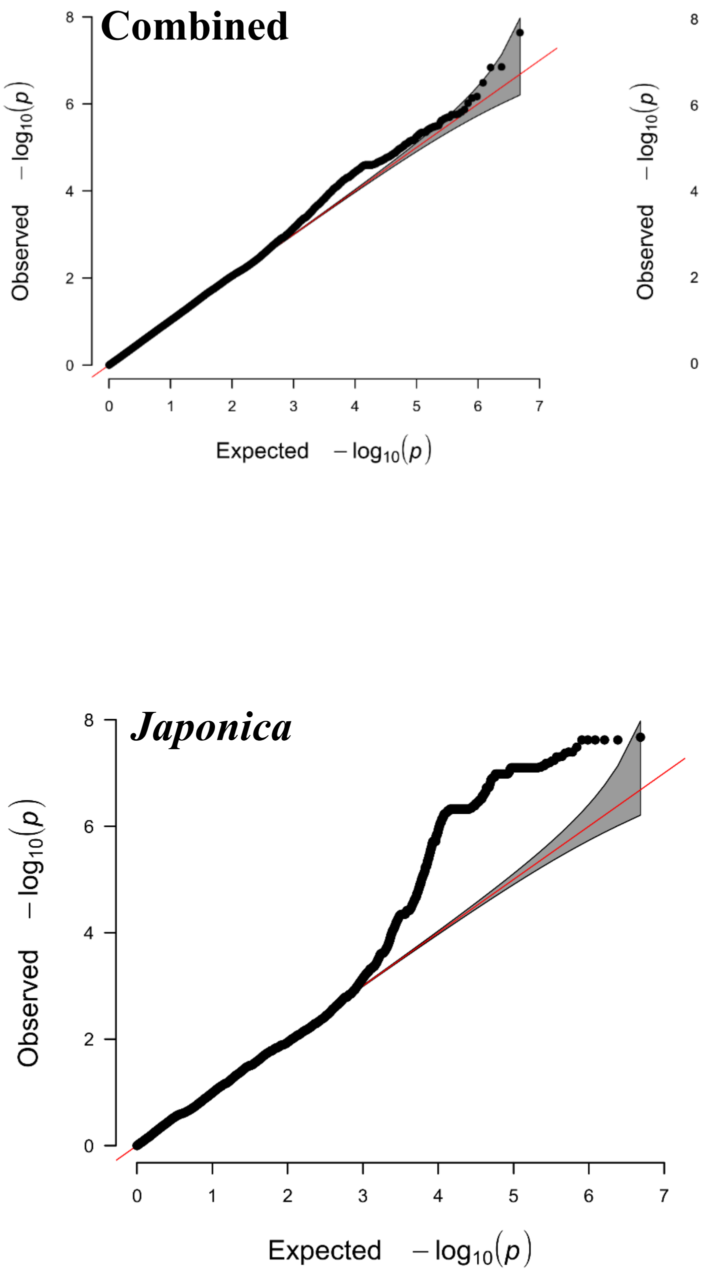

Supplementary Figure 5| Quantile-quantile (Q-Q) plots of genome-wide association studies (GWAS) results for panicle length (cm) under high nighttime temperature (HNT) stress in the Combined, *Indica*, and *Japonica* populations. The plots show the observed *p*-values (*p*) for association between panicle length (cm) and each tested marker, expressed as -log10 of the observed *p*- values (y-axis) plotted against -log10 of the expected p-values (x-axis) under the null hypothesis of no association for the analyses.

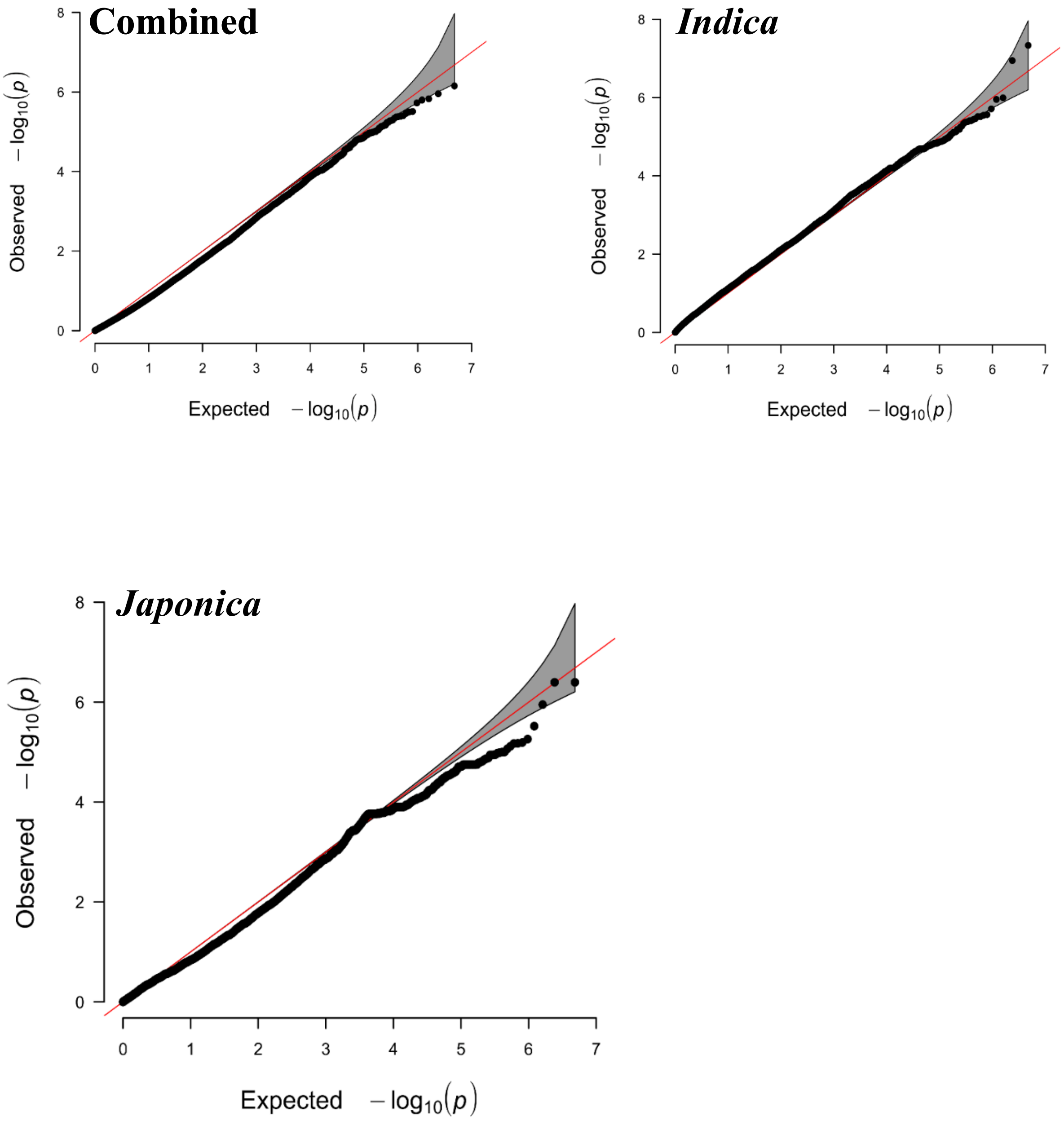

Supplementary Figure 6| Quantile-quantile (Q-Q) plots of the genome-wide association study (GWAS) results for number of spikelets per panicle under high nighttime temperature (HNT) stress in the Combined, *Indica*, and *Japonica* populations. The plots show the observed *p*-values (p) for association between number of spikelets per panicle and each tested marker, expressed as -log10 of the observed *p*- values (y-axis) plotted against -log10 of the expected p-values (x-axis) under the null hypothesis of no association for the analyses.

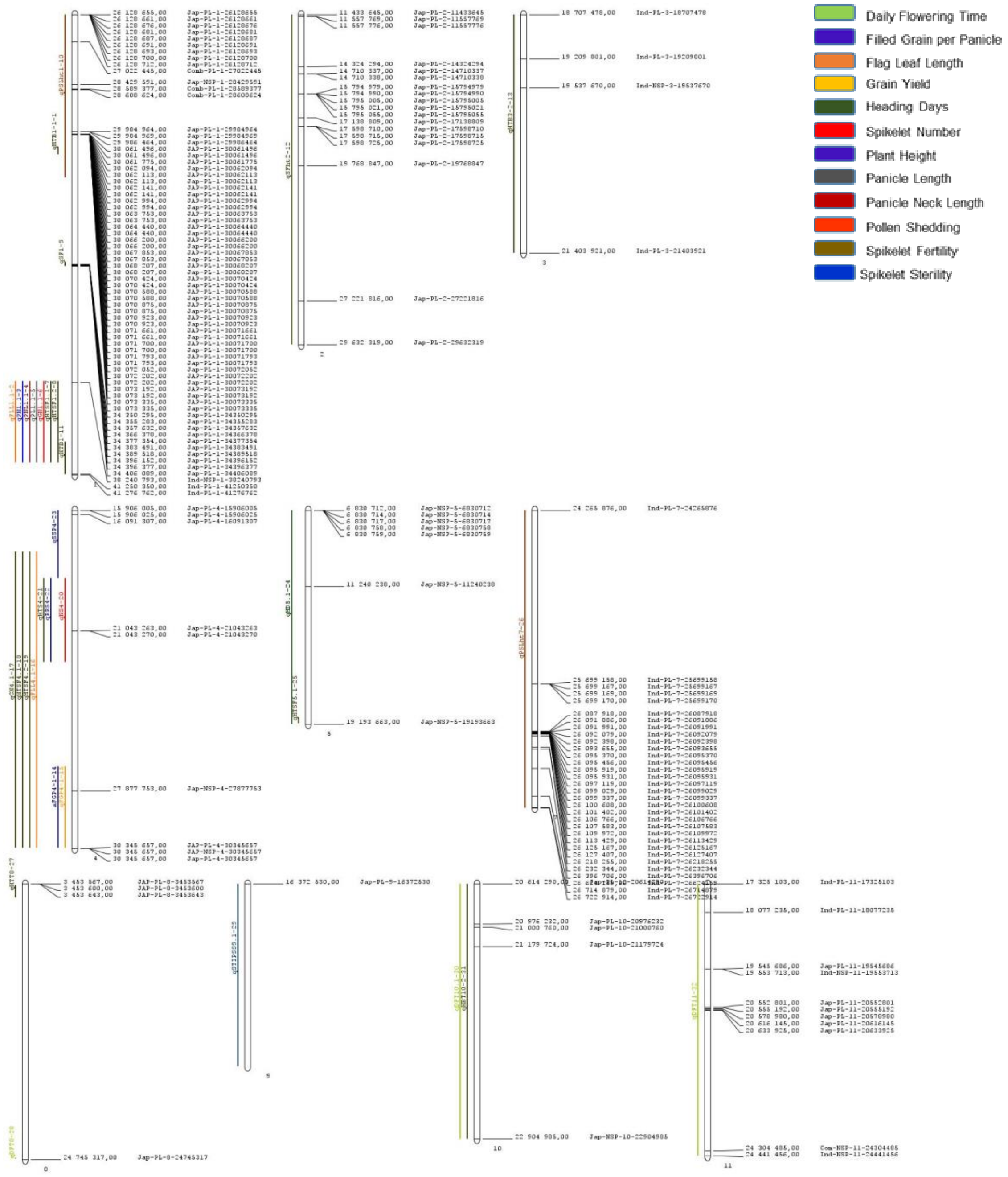

Supplementary Figure 7| Comparative physical map of the highly significant genome-wide SNPs/loci (positions in cM) associated with panicle length and number of spikelets per panicle in the Combined, *Indic*a, and *Japonica* populations under high nighttime temperature (HNT) stress (in the present study), and the previously reported quantitative trait loci (QTLs) for grain components under heat stress in rice. On each chromosome, the highly significant GWAS SNP positions (in bp) are shown on the right-hand side, along with previously reported QTLs and their annotation highlighted on the left-hand side. Each QTL for individual grain yield component is represented by an independent color as highlighted.

A

B

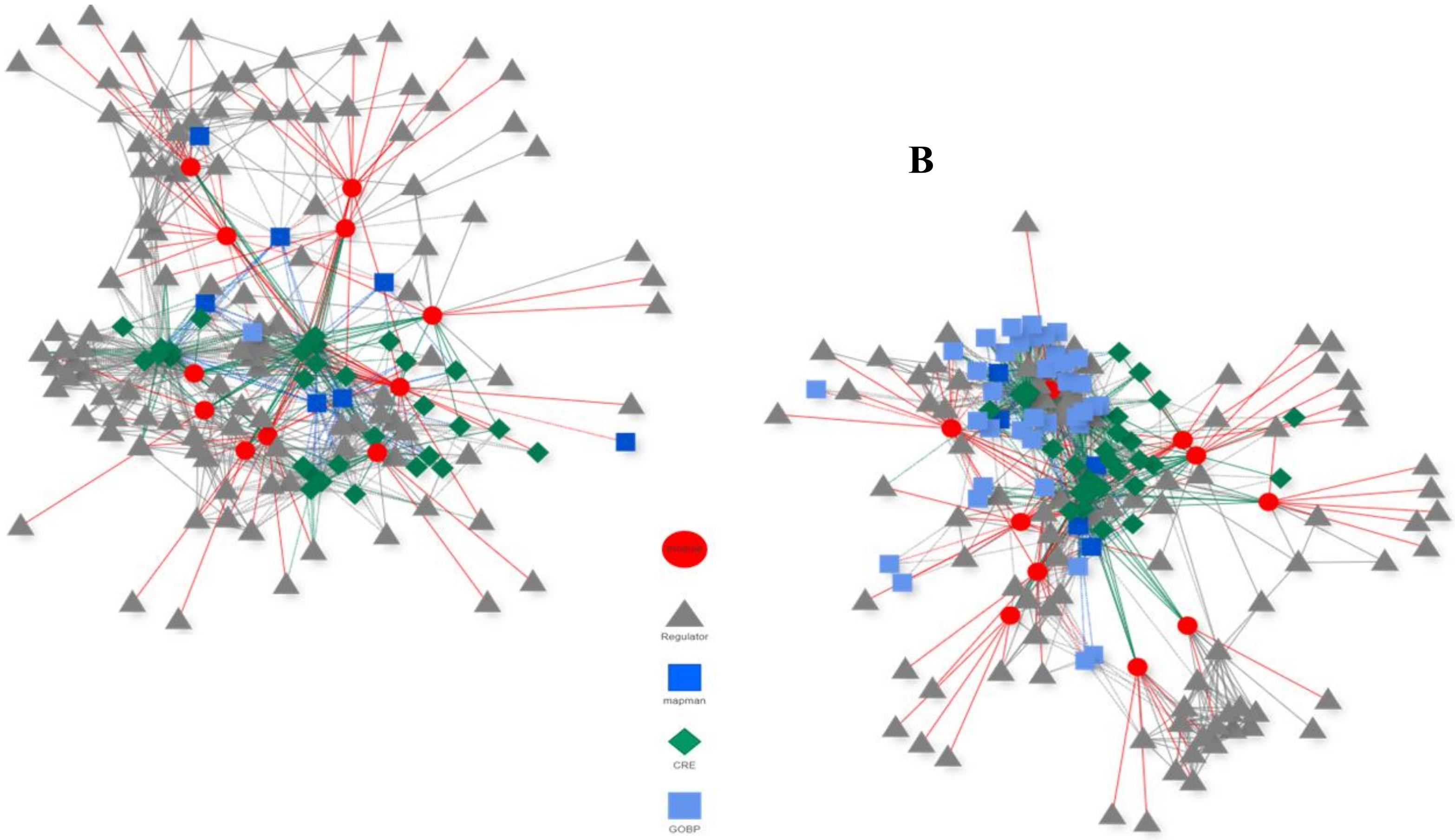

Supplementary Figure 8| Networks of panicle length (PL) and number of spikelets per panicle (NSP). Network analysis was performed with selected top SNPs from GWAS under HNT stress. **(A)** For the PL gene-list, the Network resolves into 11 significantly enriched modules of functionally related and co-regulated genes, comprised of stress-related genes, signaling kinases and genes involved in post translational modification as annotated by the Mapman tool, and potential biological processes following rice Gene Ontology annotation. **(B)** Using the NSP gene-list, GRAiN identified 10 modules of functionally related and co-regulated genes, mainly involved in different categories of carbohydrate metabolism, and signaling, and DNA repair pathways. The network symbols describe the Modules (*Red circle)*, GO biological process (*light blue square*), Mapman pathway (*dark blue square*), cis-regulatory elements-CREs (dark green diamond), and top 10 regulators “transcription factors” (*gray triangle*). The network represents the top 5% significant edges of the data.
